# Life‐History, Stress‐Resistance, and Transcriptomic Variation Among a Mexican *Caenorhabditis elegans* Isolate and Contemporary and Ancestral N2 Lineages

**DOI:** 10.1002/ece3.74378

**Published:** 2026-09-30

**Authors:** Jaqueline Hersch‐González, Nallely Cano‐Domínguez, Mariana Zurita‐León, Francisco Pinta‐Castro, Maribel Soto‐Nava, Santiago Avila‐Rios, Victor Julian Valdes

**Affiliations:** ^1^ Departamento de Biología Celular y Desarrollo, Instituto de Fisiología Celular (IFC) Universidad Nacional Autónoma de México (UNAM) Ciudad de México México; ^2^ Centro de Investigación en Enfermedades Infecciosas (CIENI) Instituto Nacional de Enfermedades Respiratorias (INER) Mexico City México

**Keywords:** *C. elegans*, domestication, heat stress, life history, N2 Ancestral, natural variation, transcriptome, wild isolate

## Abstract

Long‐term laboratory propagation can be associated with phenotypic and transcriptional differences in model organisms, but comparisons with recently isolated strains and earlier laboratory lineages remain limited. Here, we compared a recently isolated *Caenorhabditis elegans* strain from Mexico City (VJV2) with the contemporary N2 Bristol strain. To contextualize laboratory propagation history, we also included the N2 Ancestral strain, a cryopreserved lineage from 1969 derived from early laboratory stocks. Phenotypic assays showed that, under standard laboratory conditions, the VJV2 strain exhibited reduced lifespan and fertility but increased survival following *Pseudomonas aeruginosa* exposure, whereas the Ancestral strain showed higher oxidative stress tolerance but reduced thermotolerance. RNA‐seq analyses showed that transcriptomic profiles of the VJV2 and Ancestral strains were more similar to each other than to the contemporary N2 Bristol strain, suggesting expression differences associated with strain history and laboratory propagation. Differential gene expression analyses identified strain‐specific expression patterns involving stress‐, immune‐, and collagen‐related genes. Under acute heat stress, only a small set of canonical heat shock genes was commonly upregulated across the three strains, whereas the VJV2, Ancestral, and contemporary N2 Bristol strains exhibited distinct strain‐specific expression patterns. Together, these findings provide a comparative phenotypic and transcriptomic characterization of three *C. elegans* strains with distinct laboratory histories and highlight the importance of strain background when interpreting experimental outcomes.

## Graphical abstract

We compared a recently isolated wild strain of *Caenorhabditis elegans* from Mexico City (VJV2) with the contemporary standard N2 Lab strain and the cryopreserved N2 Ancestral lineage. The strains differed in lifespan, fertility, and survival under pathogen, oxidative, and heat stress. Ancestral and VJV2 transcriptomes under both control and heat‐stress conditions were more similar to each other than to N2 Lab, suggesting expression differences associated with strain history and laboratory propagation.

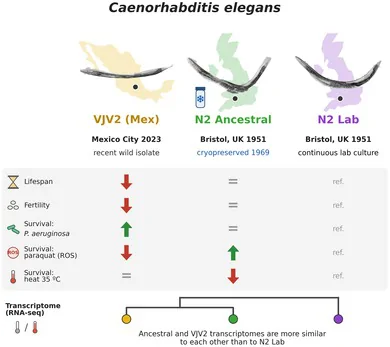

## 1 Introduction

The free‐living nematode *Caenorhabditis elegans* has been an extraordinary model organism for over 70 years, with landmark contributions to neuronal connectivity, longevity, apoptosis, microRNAs, and RNA interference, among others (Ellis and Horvitz 1986; White et al. 1986; Kenyon et al. 1993; Wightman et al. 1993; Lee et al. 1993; Fire et al. 1998).

*Caenorhabditis elegans* was first identified in Algeria in 1900 by Emile Maupas and later studied in the 1940s by Victor Nigon and Ellsworth Dougherty (Maupas 1900; Nigon and Félix 2017). However, the widespread adoption of *C. elegans* as a model organism was largely driven by Sydney Brenner, who in the 1960s started working with the genetics of a *C. elegans* strain isolated in 1951 in Bristol, UK, that he later designated as the “N2 Bristol” strain (Brenner 1974; Sterken et al. 2015). Today, hundreds of laboratories worldwide use *C. elegans* as a model system, with the N2 Bristol strain serving as a reference that has been extensively characterized and genetically manipulated (Ankeny 2001). In addition to the contemporary N2 Bristol strain, an earlier N2 lineage is available through the Caenorhabditis Genetics Center: the “Ancestral” N2 stock, derived from material cryopreserved in 1969 and estimated to be fewer than six generations from that frozen stock (Sterken et al. 2015).

Part of the success of *C. elegans* as a model organism is based on its easy culture and maintenance: it grows on agar plates at 20°C (±5°C) with *E. coli* as a food source (Corsi et al. 2015). These standardized laboratory conditions have been implemented by most laboratories worldwide. However, they differ significantly from the complex and dynamic natural environments that *C. elegans* encounters in the wild that include fluctuations in temperature, oxygen, and humidity, along with scarce yet diverse food sources within a three‐dimensional habitat where it faces pathogens and predators. These ecological differences make recently isolated wild strains valuable for examining phenotypic and transcriptional variation outside the standard N2 laboratory background.

Given the short‐life cycle of *C. elegans* (one generation every 72 h at 20°C), thousands of generations of the N2 Bristol strain have likely been propagated under laboratory conditions since its isolation more than 70 years ago. Laboratory adaptation, domestication, and unintended genetic drift have therefore been proposed to contribute to divergence in the N2 Bristol strain (Weber et al. 2010; Sterken et al. 2015). The recognition of this divergence has motivated large‐scale efforts to characterize natural diversity in *C. elegans* . Early work from the Félix lab was central in establishing the ecological and geographic context of wild isolates, forming the foundation for what later became the *C. elegans* Natural Diversity Resource (CaeNDR) (Barrière and Félix 2005; Cook et al. 2017). In recent years, efforts have been made to isolate and study different wild isolates from diverse geographical locations. For instance, the CaeNDR currently catalogs over 2000 wild isolates collected from every continent except Antarctica (Crombie et al. 2024). To complement these collections, studies have examined phenotypic, genomic, and transcriptomic variation across hundreds of isolates. Zhang et al. (2022) sequenced the transcriptomes of 207 genetically distinct wild strains, providing one of the most comprehensive datasets of expression diversity in the species and highlighting the extent of transcriptional plasticity across natural isolates.

Extensive work has also shown that a substantial fraction of transcriptomic variation across *C. elegans* isolates is genetically controlled. Expression quantitative trait locus (eQTL) studies have revealed both cis‐ and transacting regulatory variants that produce widespread expression differences and regulatory hotspots affecting hundreds of genes (Rockman et al. 2010; Snoek et al. 2017). More recently, large‐scale analyses have further supported genetic background as an important predictor of expression profiles, including across environmental conditions (e.g., Bell et al. 2025).

Many recently isolated strains exhibit genotypic and phenotypic differences compared to each other and to the reference N2 Bristol strain (Weber et al. 2010; Sterken et al. 2015; Zhao et al. 2018; Urban et al. 2021). One illustrative example of natural variation in behavior is the “social” phenotype observed in a Hawaiian isolate and other wild strains, which is associated with a different isoform at the NPR‐1 neuropeptide Y receptor. These strains carry a phenylalanine at position 215, whereas the standard N2 Bristol strain has a valine at this position. This variant leads to a preference for aggregation of individuals under high‐oxygen laboratory conditions, in contrast to the “solo” feeding behavior displayed by the N2 Bristol strain on the agar plates (de Bono and Bargmann 1998; Gray et al. 2004; Sterken et al. 2015).

While the domestication of N2 has been previously documented, most studies have focused either on genetic differences among laboratory substrains or on broad comparisons between N2 and wild isolates. Less attention has been given to simultaneous comparisons of phenotype and gene expression across the contemporary N2 Bristol strain, the earlier N2 Ancestral lineage, and recently collected wild isolates. Such comparisons can provide a useful framework to describe how laboratory history and recent isolation are associated with phenotypic and transcriptional variation.

In this study, we compared life‐history traits, stress‐resistance phenotypes, and gene expression profiles among three *C. elegans* strains: (i) a N2 Bristol strain recently acquired from the CGC (N2 Lab), (ii) the N2 Ancestral strain, also from the CGC, and (iii) VJV2, a wild strain recently isolated in Mexico City. We found that the strains respond differently to biotic and abiotic stressors, exhibiting differences in lifespan and fertility. We also observed that the N2 Lab strain displays the most distinct transcriptomic profile, whereas the Ancestral and VJV2 strains are more similar to each other. Together, these findings provide a comparative characterization of three *C. elegans* strains with distinct laboratory histories and highlight the importance of considering strain background when interpreting experimental outcomes.

## 2 Results

### 2.1 Isolation and Characterization of a Wild *Caenorhabditis elegans* Isolate From Mexico City

While Bristol, UK, is a coastal city with a humid cold climate (2°C–25°C; average 11°C), Mexico City is located over 2000 m above sea level characterized by well‐defined dry and wet seasons and a warmer temperature (8°C–34°C; average 20°C). To compare the N2 Bristol strain with a recent *C. elegans* isolate, we collected samples from rotting fruit in gardens across Mexico City, obtaining selfing worm populations. Microscope observation revealed some individuals with the distinctive double‐bulb pharynx characteristic of *C. elegans* (Figure 1A) that were propagated by self‐fertilization. We confirmed the species identity using ITS2 PCR amplicons and Sanger sequencing of a 777 bp product, which confirmed the identity of the Mexican strain as *C. elegans* (Figure S1A,B). To further confirm species identity, we backcrossed this new VJV2 (Mexican) isolate with males from the *C. elegans* OH10689 strain (carrying a neuronal RFP reporter), obtaining fluorescent, self‐propagating progeny (data not shown). The VJV2 (Mexican) strain was cultured under standard laboratory conditions, and several vials were frozen to minimize laboratory propagation. For the experiments in this study, we employed worms within the first 10 generations derived from these original frozen stocks. The Mexican strain was registered as VJV2 in CaeNDR. In the rest of the text, it will be referred to as the “Mexican strain.”

**FIGURE 1:**
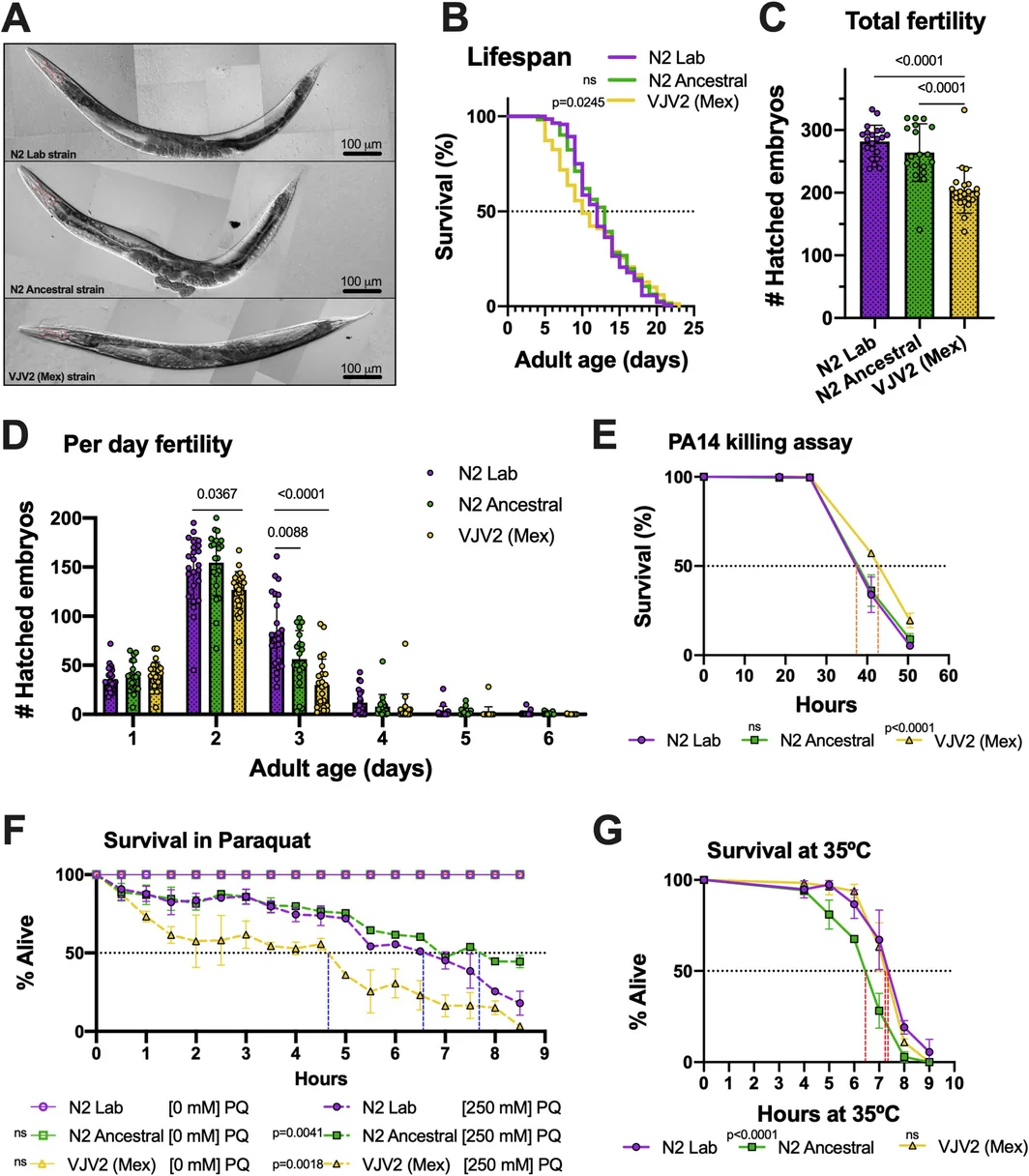
Phenotypic characterization of N2 Lab, N2 Ancestral, and VJV2 (Mex) *C. elegans* strains. (A) Representative images showing similar morphology across strains. The red dotted lines show the distinctive double‐bulb pharynx characteristic of *C. elegans* . (B) Lifespan analysis. Data are mean values of three plates (~60 worms each). Worms that exhibited bagging or left the plate were censored. Statistical significance was assessed using the Gehan–Breslow–Wilcoxon test, comparing each strain to N2 Lab. *N* indicates the total number of worms included in the analysis after censoring (N2 Lab = 140; N2 Ancestral = 142; VJV2 (Mex) = 149). Median lifespans: N2 Lab = 12 days; N2 Ancestral = 13 days; VJV2 (Mex) = 10 days. (C, D) Total and daily fertility. Hatched embryos were counted daily for 6 days post‐L4, one nematode per plate (day 0 = L4 stage). Total fertility values (sum of offspring over 6 days): N2 Lab = 281.6; N2 Ancestral = 264.1; VJV2 (Mex) = 203.6. Total nematodes used: N2 Lab = 21; N2 Ancestral = 18; VJV2 (Mex) = 22. Significance was determined by one‐way ANOVA with multiple comparisons. (E) Survival curves following *Pseudomonas aeruginosa* PA14 exposure. The time to 50% lethality (LT50): N2 Lab = 37 h; N2 Ancestral = 37 h; VJV2 (Mex) = 43 h. Three plates per strain were used, each with a PA14 lawn and ~60 worms per plate. Hour 0 corresponds to the L4 stage. Significance was assessed by the Log‐rank (Mantel‐Cox) test comparing each strain to N2 Lab. (F) Survival curves under oxidative stress (paraquat). ~30 L4 worms were placed in wells containing M9 medium with *E. coli* and paraquat at [250 mM] or [0 mM] for controls, with duplicates per strain. LT50: N2 Lab = 6.6 h, N2 Ancestral = 7.8 h, and VJV2 (Mex) = 4.7 h. Significance was determined using the Log‐rank (Mantel‐Cox) test comparing each strain to N2 Lab. (G) Survival curves under heat stress (35°C). LT50: N2 Lab = 7.5 h; N2 Ancestral = 6.5 h; VJV2 (Mex) = 7.5 h. ~25 day 1 adult (A1) worms per plate, with three replicates per strain per hour, were exposed to a constant temperature bath at 35°C. Significance was assessed by Log‐rank (Mantel‐Cox) test comparing each strain to N2 Lab. All graphs show representative experiments; the lifespan and 35°C assays were performed in three independent biological replicates, and the PA14 and paraquat assays in two.

Initial observations revealed that the Mexican strain displayed aggregation at the edges of the bacterial lawn, consistent with the “social feeding” phenotype (Figure S1C), a region reported to have a lower oxygen concentration (Gray et al. 2004). Several wild *C. elegans* isolates share this phenotype, which has been associated with the NPR‐1 neuropeptide Y receptor variant 215F (de Bono and Bargmann 1998). The presence of the “social” allele in the Mexican strain was corroborated by Sanger sequencing (data not shown). In parallel, we obtained a sample of the CGC's N2 “Ancestral” strain, which—although propagated under laboratory conditions—is estimated to be within six generations of the original frozen stock, and it was compared to the standard CGC's N2 (var Bristol) strain obtained by us in 2023, here referred to as “N2 Lab.” Under our conditions, both the N2 Lab and Ancestral strains displayed solitary feeding. These three strains allowed us to compare a recent Mexican isolate, an early N2 Bristol laboratory lineage and the contemporary N2 Bristol strain.

### 2.2 The Mexican Strain Exhibits Reduced Lifespan and Fertility Under Laboratory Conditions

It has been reported that *C. elegans* wild isolates often exhibit lifespan differences compared to the N2 Bristol reference strain (Lee et al. 2016). Even among N2 maintained in different laboratories, variability in lifespan has been reported (Urban et al. 2021). Therefore, we evaluated the lifespan of the three strains, finding that while the N2 Lab and Ancestral strains showed no statistically significant difference, having a median survival of 12 days (*n* = 140) and 13 days (*n* = 142), respectively, the Mexican strain had a shorter median lifespan of 10 days (*n* = 149, *p* = 0.0245) (Figure 1B). To our knowledge, there are only two previous mentions regarding the lifespan of the Ancestral strain: one reported it to be shorter than the reference N2 (D. Gems, personal communication, Worm Breeder's Gazette 16(5):24, 2001), while the other found no differences between the two (Lee et al. 2016). Our observations indicate that the Mexican strain has a reduced lifespan under standard laboratory conditions.

Fertility is another key life‐history trait that reflects important aspects of an organism's biology, and reduced fertility in other wild isolates has also been reported (Gutteling et al. 2007; Zhang et al. 2021). We therefore evaluated both total and day‐by‐day fertility, finding significant differences among the three strains (Figure 1C,D). Daily fertility analysis showed that the Mexican strain had reduced offspring production on the second day compared to both N2 strains (N2 Lab = 146.4; N2 Ancestral = 154.6; Mexican = 126.7, adjusted *p*‐value = 0.0367), while on the third day, both the Ancestral and Mexican strains produced fewer offspring than the N2 Lab strain (Figure 1D, N2 Lab = 83.75; N2 Ancestral = 56.25, adjusted *p*‐value = 0.0088; Mexican = 30, adjusted *p*‐value < 0.0001). The total fertility, measured as the sum of offspring produced over 6 days, was highest in N2 Lab (281.6, *n* = 21), followed by the Ancestral (264.1, *n* = 18), and lowest in the Mexican strain (203.6, *n* = 22, adjusted *p*‐value < 0.0001). There is a previous mention of the Ancestral strain having reduced fertility compared to the N2 Lab strain (260 vs. 292) (D. Gems, personal communication, Worm Breeder's Gazette 16(5):24, 2001).

The Ancestral strain also exhibited lower fertility at day 3 compared to the N2 Lab strain. This difference further illustrates phenotypic variation between these two N2 lineages under our experimental conditions. In this regard, studies on N2 domestication have shown that the laboratory‐derived *nath‐10* allele that both N2 strains have correlates with increased brood size by enhancing sperm production, providing a selective advantage under laboratory conditions (Duveau and Félix 2012; Sterken et al. 2015). By contrast, the lower fertility observed in the Mexican strain may be attributable to multiple factors such as differences in sperm production, oocyte quality, or embryonic viability.

Because natural variation in RNA interference (RNAi) sensitivity has been extensively documented among wild *C. elegans* isolates and shown to depend on genetic background and domestication history (Elvin et al. 2011; Chou et al. 2024), we assessed RNAi responsiveness across strains as part of this baseline characterization. No major differences were observed among the three strains in RNAi responsiveness or chemotaxis to the tested odorants (Figure S1D,E), indicating that subsequent phenotypic and transcriptional comparisons are unlikely to be confounded by strain‐specific defects in RNAi competence or broad sensory perception.

Overall, these results show that the Mexican strain has reduced lifespan and fertility under standard laboratory conditions, while the Ancestral strain shows a more modest reduction in fertility relative to N2 Lab.

### 2.3 Strain‐Specific Differences in Resistance to Biotic and Abiotic Stressors

In its natural environment, nematodes face pathogen threats. To investigate how the studied strains respond to biotic stress, we evaluated the survival following infection with *Pseudomonas aeruginosa* strain PA14. The Mexican strain exhibited increased survival following PA14 exposure, with 50% lethality occurring at 43 h postinfection (hpi), compared to the median lethal time of 37 hpi for both the N2 Lab and Ancestral strains (Figure 1E). This result indicates that the Mexican strain is more resistant to PA14 under the conditions tested.

In its natural environment, *C. elegans* can also encounter abiotic stressors such as oxidative stress caused by heavy metals and microorganisms (Martinez‐Finley and Aschner 2011; Kniazeva and Ruvkun 2019; Mattila et al. 2022). Therefore, we exposed the different strains to paraquat (PQ) to evaluate the strain‐specific differences in oxidative stress resistance. Figure 1F shows that the Mexican strain was the most susceptible to PQ, with a median lethal time (LT_50_) of 4.7 h, nearly 2 h earlier than the N2 Lab strain (LT_50_ = 6.6 h). The Ancestral strain was the most resistant, with a LT_50_ of 7.8 h.

Another abiotic stressor we evaluated was temperature. We exposed adult worms to 35°C and monitored their survival every hour. The Mexican and N2 Lab strains exhibited similar survival profiles, with a lethal median time (LT_50_) of 7.5 h, whereas the Ancestral strain was more susceptible to heat stress with a LT50 of 6.5 h (Figure 1G). The Mexican strain comes from a warmer environment, where temperatures range from 8°C to 34°C, the highest registered temperature in Mexico City; while Bristol (UK), origin of the N2 strain, experiences a colder climate, ranging from 2°C to 25°C. Although these geographic and laboratory histories provide useful context, the mechanisms underlying the observed heat‐stress differences remain to be investigated. It would be informative to assess the survival under less acute heat‐stress conditions to better understand how these strains differ in thermal tolerance.

Together, these results indicate that each strain exhibits distinct stress‐resistance profiles under the conditions tested: the Mexican strain showed increased survival following PA14 exposure but is more vulnerable to oxidative stress; the Ancestral strain showed heightened oxidative stress tolerance but reduced thermotolerance and pathogen resistance; and the N2 Lab strain showed intermediate oxidative stress and temperature tolerance.

### 2.4 Transcriptomic Profiles Reveal Greater Similarity Between N2 Ancestral and Mexican Strains

The observed phenotypic variability of life‐history traits among the three strains may arise from multiple sources including underlying genetic variation as well as differences in regulatory responses. To explore transcriptional differences, we analyzed transcriptomic profiles by RNA‐seq under standard conditions. Principal component analysis (PCA) showed tight clustering of replicates, indicating high intra‐strain consistency. However, the N2 Lab strain clustered distinctly from the other strains along PC1, which accounted for 78% of the total variance. In contrast, the Mexican and Ancestral strains were separated primarily along PC2, explaining 13% of the variance (Figure S2A). Together, these results indicate greater transcriptional divergence between the N2 Lab strain and the Ancestral and Mexican strains than between the latter two. This pattern is consistent with previous studies showing that the Bristol N2 strain exhibits strain‐specific transcriptomic profiles and clusters apart from other isolates (Lansdon et al. 2022).

Because all RNA‐seq reads were aligned to the Bristol N2 reference genome, we evaluated whether sequence divergence could introduce mapping bias that might influence global expression patterns. Under control conditions, uniquely mapped read percentages were comparable among strains (N2 Lab, 87.7%–88.2%; N2 Ancestral, 85.5%–87.6%; Mexican, 77.5%–86.4%), with multi‐mapping rates below 5% in all samples (Table S1). These values indicate no major impairment of read alignment due to sequence divergence and support the interpretation that the observed PCA structure largely reflects biological transcriptional differences rather than major technical artifacts. Any residual mapping bias would be expected to conservatively reduce apparent expression levels in divergent strains and therefore cannot readily explain the greater similarity observed between the Mexican and Ancestral strains relative to N2 Lab. Nonetheless, incorporating strain‐specific genomic references in future analyses will be important to further refine these results.

Following the PCA, we performed differential gene expression (DEG) analyses comparing both Mexican and Ancestral strains against N2 Lab, using a cutoff of FDR‐adjusted *p*‐value < 0.05 and log_2_ fold change (LFC) ≥ 2. This revealed a total of 3400 DEGs across both comparisons, with 55% (1873 genes) shared between the Ancestral and Mexican strains (611 downregulated, 1262 upregulated) (Figure 2A,B). This overlap was significantly greater than expected by chance (hypergeometric test, *p* < 2.2 × 10^−16^), indicating substantial overlap in transcriptional divergence of the Ancestral and Mexican strains relative to N2 Lab. Heatmap analysis further supports that the expression profiles of these two strains are at some degree different from that of the N2 Lab strain, with DEGs representing approximately 28% of all expressed transcripts (3400/12,191) (Figure 2C). This transcriptional divergence between the N2 Lab strain and the Ancestral and Mexican strains aligns with previous reports highlighting significant gene expression shifts in wild isolates and in response to environmental factors such as diet (MacNeil et al. 2013; Snoek et al. 2017; Zhang et al. 2022).

**FIGURE 2:**
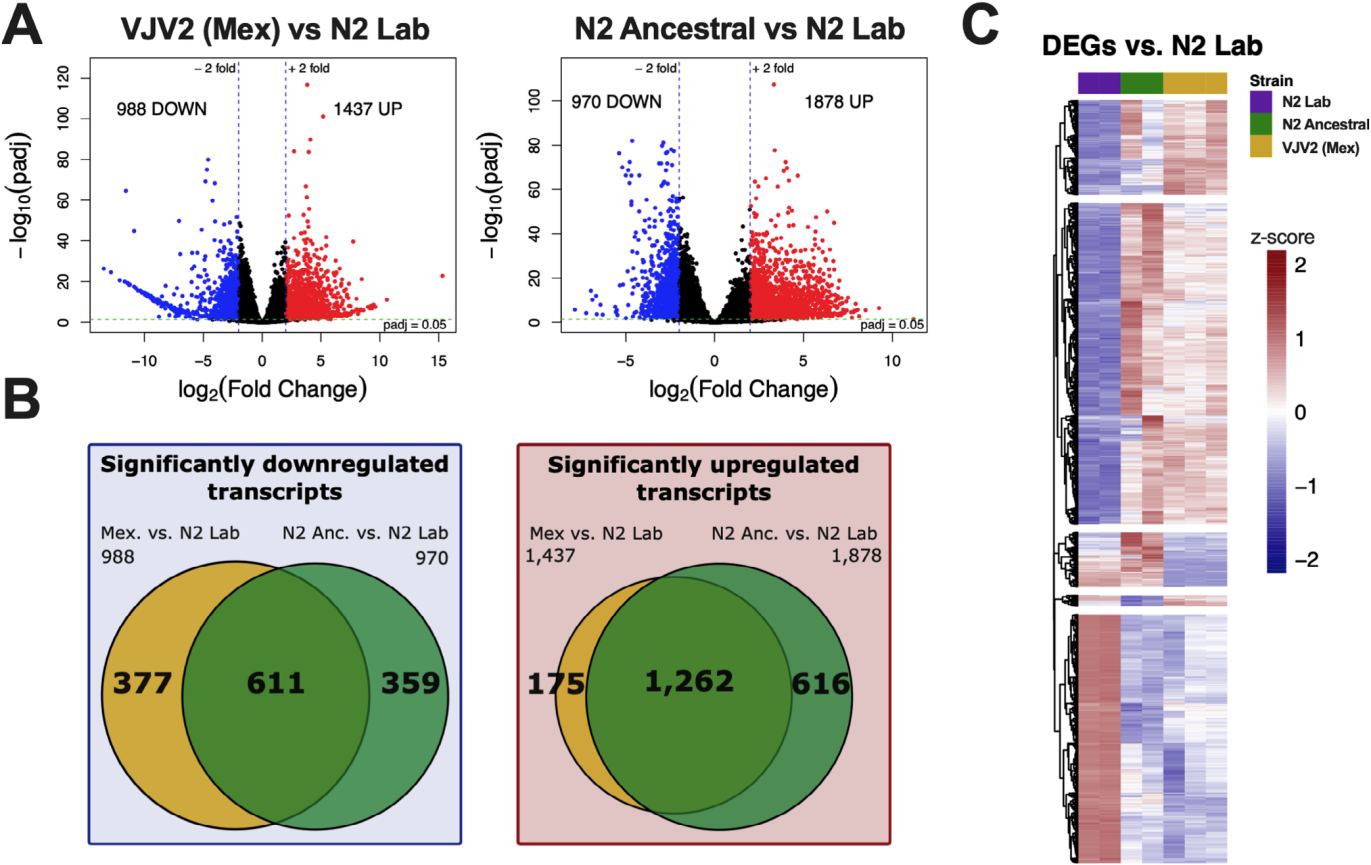
Differential gene expression in VJV2 (Mex) and N2 Ancestral relative to the N2 Lab strain. (A) Volcano plots showing differentially expressed genes (DEGs) of VJV2 (Mex) and Ancestral strains compared to N2 Lab strain. DEGs were defined as those with FDR‐adjusted *p*‐value < 0.05; log_2_ fold change (LFC) ≥ 2. (B) Venn diagrams showing the overlap of upregulated and downregulated transcripts relative to the N2 Lab strain. VJV2 (Mex) and N2 Ancestral strains share 61.5% (1262/2053) of upregulated and 45.4% (611/1347) of downregulated transcripts. (C) Heatmap for the total 3400 DEGs vs. N2 Lab strain. All DEGs have a *p*‐adjusted value ≤ 0.05 and a LFC ≥ 2.

We also examined known regulators of transcriptional variability in the Ancestral and Mexican strains. Both *hil‐2* (a linker histone cofactor) and *ttx‐1* (a thermosensory transcription factor), previously implicated in gene expression differences among wild strains, were upregulated in Mexican and Ancestral strains (Table S2), consistent with the distinct transcriptional profiles observed in these strains (Zhang et al. 2022).

### 2.5 Upregulated Genes Relate to Immune System and Pathogen Response

Venn diagram‐based grouping of DEGs allowed us to identify strain‐specific and shared expression patterns; Gene Ontology (GO) enrichment analyses were then conducted for each group (Table 1). Among Mexican‐only upregulated genes, GO terms such as “Stress response: pathogen” and “Proteolysis general: lysozyme” emerged, consistent with the increased PA14 survival observed in this strain (Figures 1E and 3A). After building a heatmap on immune system‐related genes, we found upregulation of *lys‐7* (Figure 3C), a lysozyme gene downregulated by PA14 during infection as a virulence strategy (Evans et al. 2008). When *lys‐7* expression is restored, *C. elegans* is rescued from mortality, highlighting its importance in defense against this pathogen (Fatin et al. 2017). Conversely, Mexican‐only downregulated genes included *spl‐2*, a sphingosine phosphate lyase whose hyperactivation increases susceptibility to PA14 (Nasrallah et al. 2023), also consistent with the enhanced PA14 resistance of this strain. The enrichment of immune‐related GO terms among Mexican‐specific upregulated genes identifies baseline immune‐related expression differences that may be relevant to this phenotype.

**TABLE 1:** Enriched Gene Ontology Terms for differentially expressed genes (DEGs) of N2 Ancestral vs. N2 Lab strain, and VJV2 (Mex) vs. N2 Lab strain.

Group	DEGs^a^	Gene Ontology Term^b^	Number of genes	Adjusted *p*‐value (Bonferroni)^c^
VJV2 (Mex) UP	175	Stress response: pathogen	11	1.62 × 10^−6^
		Proteolysis general: lysozyme	3	3.02 × 10^−3^
N2 Ancestral UP	616	Proteolysis proteasome: E3	47	2.67 × 10^−12^
		Stress response: detoxification	21	4.76 × 10^−7^
		Extracellular material: collagen	18	9.73 × 10^−6^
		Proteolysis general: inhibitor	9	1.26 × 10^−3^
		Stress response: pathogen	15	1.30 × 10^−3^
N2 Ancestral & VJV2 (Mex) UP	1262	Extracellular material: collagen	64	1.75 × 10^−31^
		Proteolysis proteasome: E3	107	2.02 × 10^−30^
		Neuronal function: synaptic function	63	5.60 × 10^−25^
		Transcription factor: homeodomain	29	9.57 × 10^−13^
		Metabolism: insulin	14	1.05 × 10^−6^
		Extracellular material: cuticlin	11	1.17 × 10^−4^
		Transcription factor: T box	9	1.40 × 10^−4^
		Transmembrane protein: unassigned	107	2.11 × 10^−3^
		Transcription factor: forkhead	6	8.57 × 10^−3^
VJV2 (Mex) DOWN	377	Proteolysis proteasome: E3	37	1.06 × 10^−12^
		Transcription: chromatin modification	10	2.86 × 10^−4^
		DNA: replication	8	3.41 × 10^−4^
N2 Ancestral DOWN	359	mRNA functions: binding	23	8.52 × 10^−19^
		Noncoding RNA: small RNA	9	2.33 × 10^−6^
		Cell cycle: chromosome dynamics	7	1.80 × 10^−3^
		DNA: repair	7	2.36 × 10^−3^
N2 Ancestral & VJV2 (Mex) DOWN	611	DNA: repair	17	6.33 × 10^−11^
		Transcription: chromatin modification	16	7.51 × 10^−7^
		Noncoding RNA: small RNA	11	2.28 × 10^−6^
		Development: general	12	2.50 × 10^−5^
		mRNA functions: processing	22	3.69 × 10^−5^
		Signaling: lipid	12	2.28 × 10^−4^
		Nucleic acid: binding	17	7.38 × 10^−4^
		Cell cycle: chromosome dynamics	9	1.28 × 10^−3^
		Signaling: small GTPase	14	3.33 × 10^−3^

*Note:* Blue color indicates downregulated genes; red color indicates upregulated genes.

^a^ Genes with a FDR‐adjusted *p*‐value of < 0.05 and a log_2_ fold change (LFC) ≥ 2.

^b^ Category 2 from WormCat.

^c^ Bonferroni‐adjusted *p*‐value derived from Fisher's exact test for enrichment, as implemented in WormCat.

**FIGURE 3:**
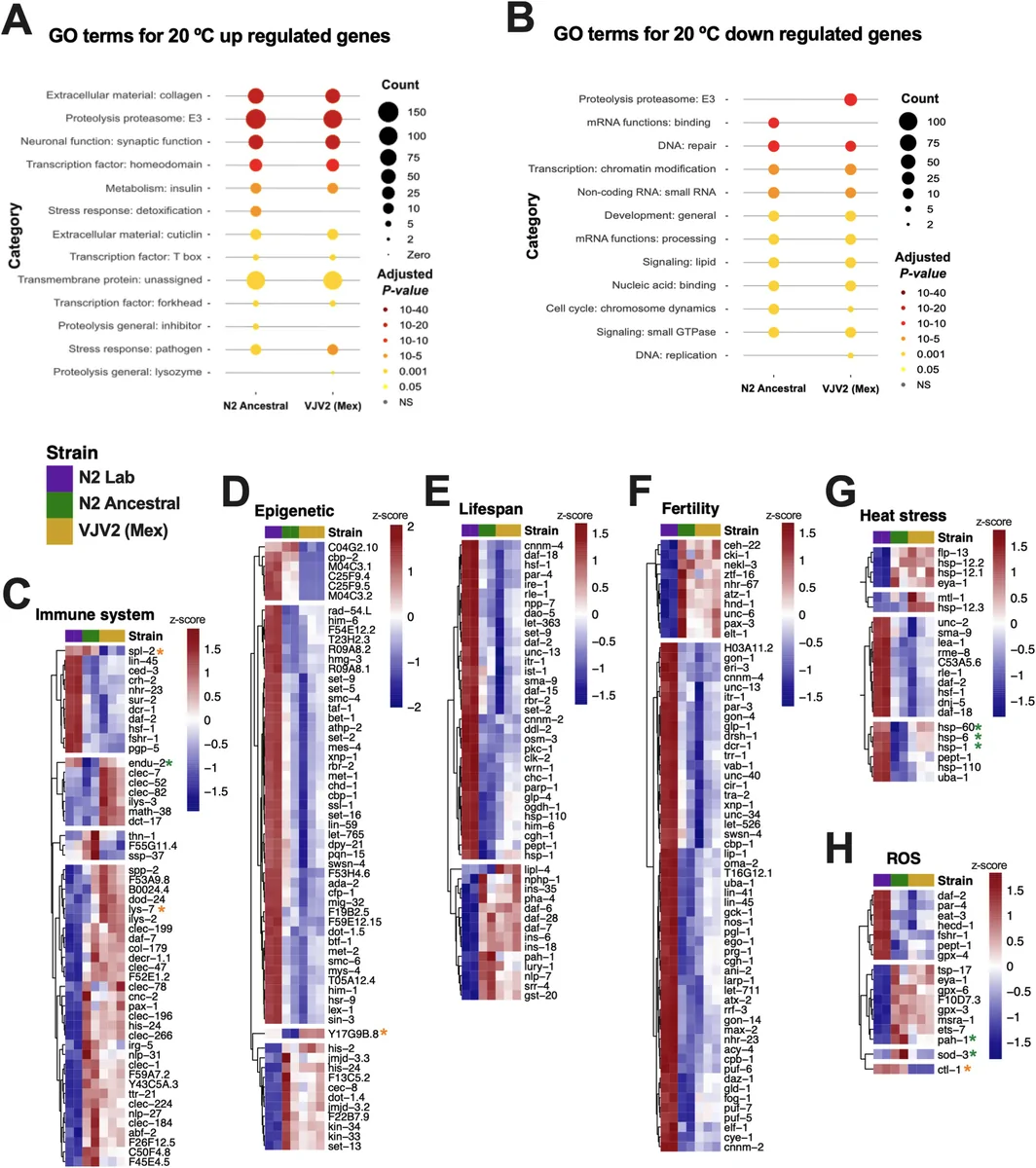
Comparative functional enrichment and differential gene expression relative to the N2 Lab strain at 20°C. (A, B) Enriched Gene Ontology Terms for up regulated (A) and down regulated (B) Ancestral and VJV2 (Mex) DEGs vs. N2 Lab strain. All terms have a Bonferroni‐adjusted *p*‐value < 0.05 (Fisher's exact test, as implemented in WormCat), indicating enrichment of the GO term among expressed genes; WormCat category = 2 (See Table 1). (C–H) Heatmaps for genes grouped by functional categories: (C) *Immune system*, (D) *Epigenetic regulation*, (E) *Lifespan*, (F) *Fertility*, (G) *Heat stress* and (H) *ROS*. All DEGs have a *p*‐adjusted value ≤ 0.05 and a LFC ≥ 2. Gene group annotations were obtained from GO terms using AmiGO (see Methods).

Within Ancestral‐only upregulated genes, terms such as “Proteolysis proteasome: E3,” “Stress response: detoxification,” “Extracellular material: collagen,” and “Stress response: pathogen” were observed, which may relate to pathogen resistance or broader environmental adaptation (Table 1 and Figure 3A). Additionally, three Ancestral‐specific immune genes appeared upregulated in the immune‐related heatmap (third cluster, Figure 3C), indicating that immune‐related expression patterns differ between the Ancestral and Mexican strains.

The shared DEGs between the Mexican and Ancestral strains included upregulation of collagen genes and terms such as “Neuronal function: synaptic function” (Figure 3A, Table 1). Variation in collagen gene expression among *C. elegans* strains has been reported (Bell et al. 2025) and is consistent with broader patterns of genetic divergence among isolates described in population‐genetic studies (Denver et al. 2004; Cutter and Ward 2005). Collagen gene expression may be relevant to strain‐specific cuticle biology, as the collagen‐rich cuticle acts as a protective barrier. Over 60 collagen transcripts were upregulated in both strains (Figure 3A, Table 1), along with upregulation of the transcription factor *elt‐3* in both strains (Table S2), which regulates collagen gene expression in response to environmental stress (Mesbahi et al. 2020). Consistent with this, ancestral cuticle properties differ from those of the N2 laboratory strain, as shown by suppression of the *rol‐1* rolling phenotype in ancestral backgrounds (Noble et al. 2020), supporting the idea that cuticle‐related traits have been shaped by laboratory adaptation. Considering that *C. elegans* possesses 173 predicted cuticular collagen genes (Teuscher et al. 2019), the extensive collagen gene upregulation observed here identifies cuticle‐related transcriptional differences shared by the Ancestral and Mexican strains.

### 2.6 Shared Mexican‐Ancestral Downregulated Genes Associate With Chromatin Remodeling

Interestingly, Ancestral and Mexican downregulated genes were enriched in “DNA: repair,” “Noncoding RNA: small RNA,” “Nucleic acid: binding,” and “Transcription: chromatin modification” (Figure 3B, Table 1). This prompted a closer examination of epigenetic regulators (after building a heatmap) where we identified six chromatin remodeling genes downregulated exclusively in the Mexican strain, alongside *Y17G9B.8*, upregulated in the Mexican strain and downregulated in the Ancestral strain (Figure 3D), which encodes the ortholog of human SGF29, a chromatin reader involved in histone H3 acetylation (Bian et al. 2011; Sternberg et al. 2024), identifying chromatin‐related genes as a potential area for future study.

### 2.7 Baseline Expression Patterns of Heat‐ and Oxidative Stress‐Related Genes Differ Across Strains

To contextualize transcriptional differences in terms of phenotypic outcomes, we explored DEGs associated with known life‐history traits such as reproduction, lifespan, and stress responses. Only one lifespan‐related gene, *lipl‐4*, was found upregulated exclusively in the Mexican strain (LFC = 1.86, adjusted *p* < 0.05; Table S2), and was therefore the only strain‐specific candidate although this did not correspond to lifespan extension (Figure 3E). Fertility‐related DEGs showed similar expression in both Ancestral and Mexican strains (Figure 3F). While *lipl‐4* upregulation is generally associated with lifespan extension (Folick et al. 2015), its lack of effect here suggests that lifespan differences cannot be explained by this gene alone.

We also examined heat‐ and oxidative stress‐related genes. The Ancestral strain showed downregulation of several heat shock genes, including *endu‐2*, *hsp‐1*, *hsp‐6*, and *hsp‐60*, which are associated with thermotolerance (Figure 3G); in particular, downregulation of *endu‐2* has been implicated in transcriptional reprogramming following heat stress (Xu et al. 2023). Using a lower threshold (LFC ≥ 1.5), we observed additional downregulated heat shock genes (*hsp‐90*, *cct‐2*, *cct‐4*, *cct‐8*, *rpn‐2*, *uba‐1*) involved in canonical heat shock response (Table S2; Guisbert et al. 2013). Regarding oxidative stress response, the Mexican strain (the most ROS‐sensitive; Figure 1F) has uniquely downregulated *ctl‐1*, a catalase critical for H_2_O_2_ detoxification (Schiffer et al. 2020; Branicky et al. 2022). In contrast, the Ancestral strain, which exhibited the highest paraquat resistance, showed upregulation of *sod‐3*, *pah‐1* (Figure 3H), and *gst‐4*, *gst‐10* and *gpx‐5* (with a lower threshold LFC ≥ 1.5; Table S2), genes linked to antioxidant defenses (Ayyadevara et al. 2005; Calvo et al. 2008; Van Raamsdonk and Hekimi 2012). Together, these findings identify strain‐specific baseline expression patterns in genes associated with environmental stress.

Finally, we directly compared gene expression of the Mexican and Ancestral strains showing that only 6.8% of the DEGs (127 down‐ and 51 upregulated, out of 2603 total DEGs) were specific between these two strains (Figure 4A,B), further supporting transcriptional similarity between these strains. GO analysis of these DEGs revealed only a few significant terms, including “Stress response: detoxification” and “Stress response: pathogen” among Mexican vs. Ancestral downregulated genes, which may reflect subtle strain‐specific adaptations (Table 2). One enriched GO term was “major sperm protein,” which was downregulated in the Mexican strain (Table 2, Figure 1C,D). These expression differences may suggest transcriptional variation in reproductive pathways that parallels phenotypic differences between strains. However, given the complexity of sperm development and fertility regulation, they should be interpreted descriptively rather than as evidence of a direct functional relationship.

**FIGURE 4:**
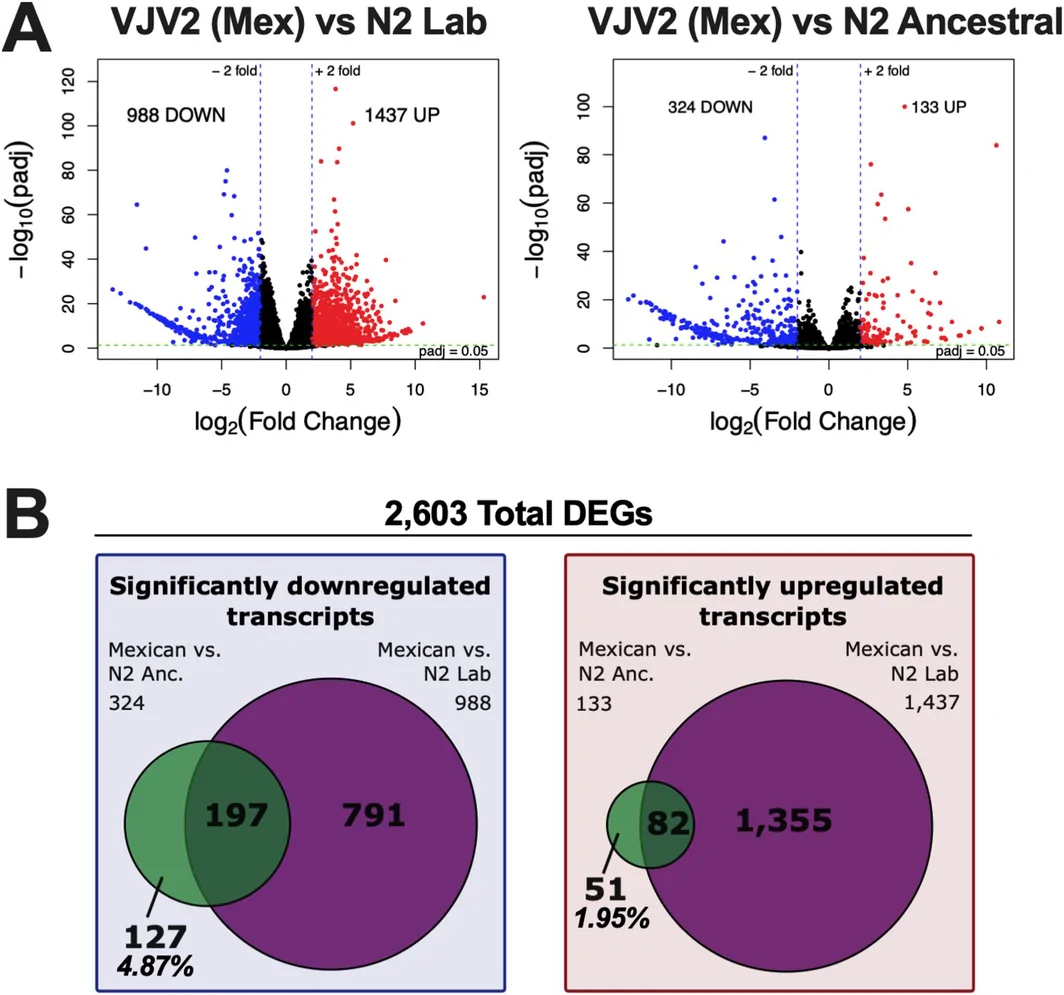
Differential gene expression in VJV2 (Mex) relative to N2 Lab and N2 Ancestral strains. (A) Volcano plots showing differentially expressed genes (DEGs) for VJV2 (Mex) vs. N2 Lab, and VJV2 (Mex) vs. Ancestral comparisons. FDR‐adjusted *p*‐value < 0.05; log_2_ fold change (LFC) ≥ 2. The VJV2 (Mex) vs. N2 Lab volcano plot is reproduced from Figure 2A to facilitate comparison. (B) Venn diagrams showing shared up‐ and downregulated transcripts between the VJV2 (Mex) strain vs. N2 Lab and vs. Ancestral strain. Of the VJV2 (Mex)‐specific DEGs relative to N2 Lab, 5.5% (82/1488) of upregulated and 17.7% (197/1115) of downregulated transcripts were also differentially expressed in the Ancestral strain.

**TABLE 2:** Enriched Gene Ontology Terms for differentially expressed genes (DEGs) of VJV2 (Mex) vs. N2 Lab strain, and VJV2 (Mex) vs. N2 Ancestral strain.

Group	DEGs^a^	Gene Ontology Term^b^	Number of genes	Adjusted *p*‐value (Bonferroni)^c^
Vs. N2 Lab UP	1355	Extracellular material: collagen	68	4.95 × 10^−33^
		Proteolysis proteasome: E3	105	5.14 × 10^−27^
		Neuronal function: synaptic function	68	6.05 × 10^−27^
		Transcription factor: homeodomain	29	5.28 × 10^−12^
		Metabolism: insulin	15	3.76 × 10^−7^
		Extracellular material: cuticlin	12	3.78 × 10^−5^
		Transcription factor: T box	9	2.54 × 10^−4^
		Signaling: hedgehog‐like	15	2.90 × 10^−3^
		Transmembrane protein: unassigned	112	3.65 × 10^−3^
		Extracellular material: secreted protein	11	7.62 × 10^−3^
Vs. N2 Ancestral UP	51	—	—	—
Vs. N2 Lab & N2 Ancestral UP	82	Proteolysis proteasome: E3	10	2.30 × 10^−4^
		Stress response: pathogen	5	5.62 × 10^−3^
Vs. N2 Lab DOWN	791	Transcription: chromatin modification	25	2.07 × 10^−12^
		DNA: repair	20	5.01 × 10^−12^
		DNA: replication	16	3.35 × 10^−8^
		mRNA functions: processing	30	1.37 × 10^−7^
		Nucleic acid: binding	26	1.93 × 10^−7^
		Noncoding RNA: small RNA	13	4.67 × 10^−7^
		Development: general	13	6.59 × 10^−5^
		Signaling: small GTPase	18	3.61 × 10^−4^
		Cell cycle: chromosome dynamics	10	1.81 × 10^−3^
		Signaling: lipid	12	3.08 × 10^−3^
Vs. N2 Ancestral DOWN	127	Proteolysis proteasome: E3	27	5.58 × 10^−17^
		Stress response: detoxification	7	9.14 × 10^−4^
		Stress response: pathogen	6	5.05 × 10^−3^
Vs. N2 Lab & N2 Ancestral DOWN	197	Proteolysis proteasome: E3	32	1.01 × 10^−16^
		Proteolysis proteasome: ubiquitin	3	1.70 × 10^−3^
		Major sperm protein	4	3.05 × 10^−3^
		Transmembrane transport: potassium channel	6	7.46 × 10^−3^

*Note:* Blue color indicates downregulated genes; red color indicates upregulated genes.

^a^ Genes with a FDR‐adjusted *p*‐value of < 0.05 and a log_2_ fold change (LFC) ≥ 2.

^b^ Category 2 from WormCat.

^c^ Bonferroni‐adjusted *p*‐value derived from Fisher's exact test for enrichment, as implemented in WormCat.

Overall, these findings highlight baseline transcriptional differences between N2 Lab and the other two *C. elegans* strains, while also supporting greater transcriptional similarity between the Ancestral and Mexican strains under standard laboratory conditions. However, because these RNA‐seq data were generated under control conditions, these patterns should be interpreted as baseline transcriptional differences rather than direct stress‐induced responses.

### 2.8 Gene Expression Patterns Under Acute Heat Stress Reveal Conserved and Strain‐Specific Transcriptional Responses

Thermal adaptation is important for organism survival; therefore, we investigated gene expression differences in response to heat stress, particularly considering the increased temperature sensitivity of the Ancestral strain (Figure 1G). To this end, animals were exposed to a sublethal heat‐stress protocol (2 h at 35°C), and RNA‐seq libraries were generated in parallel for all strains.

Principal component analysis (PCA) revealed that biological replicates clustered by strain, indicating high intra‐strain consistency. As observed under control conditions, the N2 Lab strain separated from the other strains along the first principal component (PC1), which accounted for 93% of the total variance, whereas the Ancestral and Mexican strains were primarily separated along PC2, explaining 5% of the variance (Figure S2B). We note that the N2 Lab strain under heat stress showed lower uniquely mapped read percentages (72.5%–77.6%) and higher multi‐mapping rates (14.6%–21.2%) than the other strains under the same conditions (84.1%–87.3% and below 5%, respectively; Table S1). Considering that all the strains and samples were handled simultaneously, and that this pattern is specific to N2 Lab under heat stress (consistent across biological replicates), it is unlikely to reflect sequence divergence or technical batch effects. Together, these results indicate that, even under acute heat stress, the global transcriptional profiles of the Ancestral and Mexican strains remain more similar to each other than to the laboratory‐adapted N2 strain.

To quantify strain‐specific heat responses, we performed differential gene expression (DEG) analyses comparing transcriptomes at 35°C versus 20°C within each strain (FDR‐adjusted *p*‐value < 0.05; |log_2_ fold change| ≥ 2). All three strains mounted substantial transcriptional responses to heat stress, with 2486 DEGs in the N2 Lab strain, 2942 in the Ancestral strain, and 2944 in the Mexican strain (Figure 5A). Consistent with canonical heat‐shock transcriptional signatures previously reported in *C. elegans* , heat shock proteins (HSPs) were prominently induced, forming a distinct expression cluster across strains (Figure 5B, second cluster).

**FIGURE 5:**
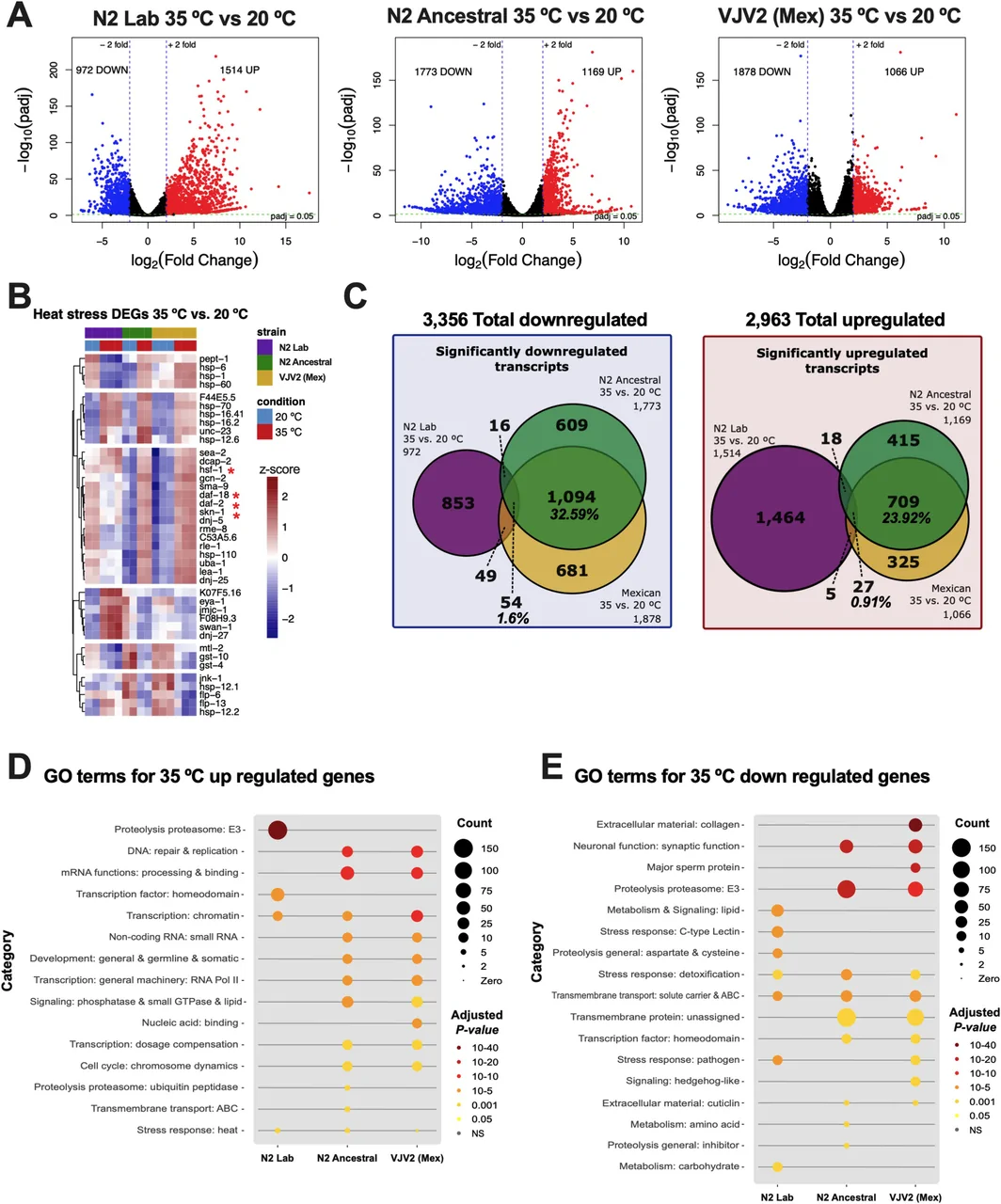
Differentially Expressed Genes in each strain under heat stress. (A) Volcano plots showing differentially expressed genes (DEGs) at 35°C compared to 20°C for each strain. FDR‐adjusted *p*‐value < 0.05; log_2_ fold change (LFC) ≥ 2. (B) Heatmap showing DEGs associated with the *Heat stress* GO term across strains. All DEGs have a *p*‐adjusted value ≤ 0.05 and a LFC ≥ 2. Gene group list was recovered from GO terms using Gene Ontology tool AmiGO (see Methods). (C) Venn diagrams showing shared up‐ and downregulated transcripts across the three strains at 35°C. Shared by the three strains: 0.91% (27/2963) upregulated; and 1.6% (54/3356) downregulated. (D) Enriched Gene Ontology Terms for upregulated DEGs at 35°C. (E) Enriched Gene Ontology Terms for downregulated DEGs at 35°C. All terms have a Bonferroni‐adjusted *p*‐value < 0.05 (Fisher's exact test, as implemented in WormCat), indicating enrichment of the GO term among expressed genes; WormCat category = 2 (See Table 3).

Despite the overall magnitude of the response, overlap of heat‐responsive genes across strains was limited. Only 0.91% (27/2963) of upregulated DEGs and 1.6% (54/3356) of downregulated DEGs were shared among all three strains (Figure 5C). Gene Ontology analysis of the 27 shared upregulated DEGs identified a single significantly enriched category, “Stress response: heat,” driven by strong induction (~8 log_2_ fold change) of *hsp‐16.2*, *hsp‐16.41*, and *hsp‐70* in all strains (Figure 5D, Table 3), underscoring their central role in the conserved heat‐shock response. These genes are among the best‐characterized heat‐inducible transcripts in *C. elegans* and have been consistently reported as strongly upregulated in heat‐shock transcriptomic studies across developmental stages and experimental conditions (Brunquell et al. 2016; Schreiner et al. 2019; Xu et al. 2023).

**TABLE 3:** Enriched Gene Ontology Terms for differentially expressed genes (DEGs) 35°C vs. 20°C.

Group	DEGs^a^	Gene Ontology Term^b^	Number of genes	Adjusted *p*‐value (Bonferroni)^c^
N2 Lab only	1464	Proteolysis proteasome: E3	176	2.85 × 10^−67^
	UP	Transcription factor: homeodomain	27	1.05 × 10^−9^
		Transcription: chromatin structure	23	1.78 × 10^−6^
		Transcription factor: bHLH	14	3.48 × 10^−6^
		Transcription factor: T box	11	1.02 × 10^−5^
		Transcription factor: forkhead	8	3.86 × 10^−4^
N2 Lab & N2 Ancestral	18	Stress response: heat	2	1.69 × 10^−3^
	UP			
N2 Ancestral only	415	Signaling: phosphatase	21	2.67 × 10^−10^
	UP	mRNA functions: binding	14	6.04 × 10^−8^
		Proteolysis proteasome: ubiquitin peptidase	6	7.71 × 10^−4^
		Transmembrane transport: ABC	6	8.68 × 10^−3^
N2 Ancestral & VJV2 (Mex)	709	DNA: repair	22	6.21 × 10^−15^
	UP	mRNA functions: processing	34	3.24 × 10^−11^
		Noncoding RNA: small RNA	15	1.28 × 10^−9^
		Development: general	17	7.24 × 10^−9^
		Transcription: chromatin modification	19	2.75 × 10^−8^
		Transcription: general machinery: RNA Pol II	18	1.05 × 10^−6^
		DNA: replication	13	3.79 × 10^−6^
		mRNA functions: binding	15	7.36 × 10^−6^
		Cell cycle: chromosome dynamics	12	1.53 × 10^−5^
		Development: somatic	15	5.31 × 10^−5^
		Signaling: small GTPase	18	8.27 × 10^−5^
		Signaling: lipid	13	2.09 × 10^−4^
		Nucleic acid: binding	17	4.97 × 10^−3^
VJV2 (Mex) only	325	Transcription: chromatin modification	16	8.63 × 10^−11^
	UP	Nucleic acid: binding	16	7.71 × 10^−7^
		DNA: replication	8	1.16 × 10^−4^
		Development: germline	5	7.62 × 10^−4^
		Transcription: dosage compensation	4	8.48 × 10^−4^
		Cell cycle: chromosome dynamics	6	7.86 × 10^−3^
		Transcription: general machinery: RNA Pol II	8	9.74 × 10^−3^
3 strains	27	Stress response: heat	3	5.02 × 10^−5^
	UP			
N2 Lab only	853	Metabolism: lipid	50	1.09 × 10^−10^
	DOWN	Stress response: C‐type Lectin	30	3.56 × 10^−8^
		Transmembrane transport: solute carrier	25	2.32 × 10^−7^
		Stress response: pathogen	24	6.25 × 10^−7^
		Proteolysis general: aspartate	11	1.88 × 10^−6^
		Metabolism: carbohydrate	13	2.01 × 10^−4^
		Proteolysis general: cysteine	9	7.33 × 10^−4^
		Transmembrane transport: ABC	9	2.96 × 10^−3^
		Stress response: detoxification	18	4.45 × 10^−3^
		Signaling: lipid	12	6.59 × 10^−3^
N2 Lab & N2 Ancestral	16	Metabolism: short chain dehydrogenase	2	2.01 × 10^−3^
	DOWN			
N2 Ancestral only	609	Proteolysis proteasome: E3	62	9.66 × 10^−22^
	DOWN	Stress response: detoxification	23	6.08 × 10^−7^
		Metabolism: amino acid	10	9.95 × 10^−4^
		Proteolysis general: inhibitor	9	1.20 × 10^−3^
		Transmembrane protein: unassigned	58	8.01 × 10^−3^
		Stress response: unassigned	7	8.25 × 10^−3^
N2 Ancestral & VJV2 (Mex)	1094	Neuronal function: synaptic function	71	2.09 × 10^−34^
	DOWN	Proteolysis proteasome: E3	76	2.38 × 10^−17^
		Transmembrane transport: solute carrier	26	6.23 × 10^−6^
		Transmembrane protein: unassigned	104	3.27 × 10^−5^
		Transcription factor: homeodomain	17	3.54 × 10^−5^
		Extracellular material: cuticlin	9	1.51 × 10^−3^
		Stress response: detoxification	21	3.97 × 10^−3^
VJV2 (Mex) only	681	Extracellular material: collagen	67	2.33 × 10^−49^
	DOWN	Major sperm protein	23	2.49 × 10^−22^
		Neuronal function: synaptic function	22	9.81 × 10^−6^
		Transcription factor: homeodomain	14	1.17 × 10^−5^
		Stress response: pathogen	19	1.86 × 10^−5^
		Signaling: hedgehog‐like	13	2.88 × 10^−5^
		Metabolism: insulin	7	4.79 × 10^−3^
VJV2 (Mex) & N2 Lab	49	Stress response: pathogen	5	4.39 × 10^−4^
	DOWN			
3 strains	54	Transmembrane transport*	8	2.99 × 10^−3^
	DOWN			

*Note:* Blue color indicates downregulated genes; red color indicates upregulated genes.

^a^ Genes with a FDR‐adjusted *p*‐value of < 0.05 and a log_2_ fold change (LFC) ≥ 2.

^b^ Category 2 from WormCat, * from Category 1.

^c^ Bonferroni‐adjusted *p*‐value derived from Fisher's exact test for enrichment, as implemented in WormCat.

In contrast, the Ancestral and Mexican strains shared a substantially larger fraction of DEGs: 23.92% (709/2963) of upregulated genes and 32.59% (1094/3356) of downregulated genes (Figure 5C), supporting their closer transcriptional similarity under heat stress. The overlap of differentially expressed genes between the Ancestral and Mexican strains under heat stress was highly significant (hypergeometric test, log_10_ *p* = −674.6), indicating a nonrandom shared set of expression changes. Shared upregulated genes were enriched for functions related to DNA repair, transcription, and signaling (Figure 5D, Table 3), whereas shared downregulated genes were enriched for neuronal function, transmembrane proteins, and proteasome‐mediated proteolysis (Figure 5E, Table 3), indicating shared functional categories among heat‐responsive genes in these two strains.

To further dissect strain‐specific expression patterns, we analyzed DEGs grouped by Venn‐diagram categories (Figure 5C). Among the few transcripts upregulated in both the Ancestral and N2 Lab strains (18 genes), only two were directly associated with heat stress: *F44E5.5 and hsp‐12.6* (Table 3). *F44E5.5* encodes an ortholog of human *HSPA6/HSPA7* and is induced by the heat shock transcription factor HSF‐1 in *C. elegans* (Brunquell et al. 2016). Both genes have been previously reported among heat‐inducible transcripts in HS transcriptomic studies, further supporting their role in the conserved stress response (Brunquell et al. 2016). Although neither gene met DEG thresholds in the Mexican strain, *hsp‐12.6* showed near‐significant induction (LFC = 1.22; adjusted *p*‐value = 0.054), suggesting a similar trend below the selected DEG threshold.

Clustering analysis further revealed strain‐specific regulatory programs (Figure 5B). In the Ancestral strain, strong induction of *hsp‐6* and *hsp‐60* was observed within a cluster that also includes *hsp‐1*. This cluster showed moderate induction in the Mexican strain but was downregulated in the N2 Lab strain. While *hsp‐1* is a core component of the heat‐shock response, *hsp‐6* and *hsp‐60* are key mediators of the mitochondrial unfolded protein response, and *pept‐1*, another gene in this cluster, encodes a transmembrane transporter (Sternberg et al. 2024). These patterns may be compatible with enhanced activation of mitochondrial stress pathways in the Ancestral and Mexican strains but would require further investigation.

Among Mexican‐specific upregulated DEGs, *hsf‐1* was the only canonical heat‐shock response regulator identified. HSF‐1 orchestrates proteostasis under heat stress by inducing molecular chaperones such as *hsp‐16* family members and *hsp‐70*, thereby promoting protein refolding and degradation of damaged proteins (van Oosten‐Hawle and Morimoto 2014; Jones et al. 2018). Notably, *hsf‐1* clustered with 15 additional genes upregulated in both the Mexican and Ancestral strains, including *dnj‐5*, *rme‐8*, *hsp‐110*, *daf‐2*, *daf‐18*, and *skn‐1*, which are implicated in stress signaling and longevity pathways (Zhang et al. 2001; Jones et al. 2018; Sternberg et al. 2024). In contrast, these genes showed little or no induction in the N2 Lab strain, despite higher baseline expression at 20°C, consistent with strain‐specific differences in heat‐induced expression patterns. A previous heat‐shock transcriptomic study has also reported downregulation of *hsf‐1* and *daf‐18* expression during prolonged heat stress in the N2 laboratory strain (Schreiner et al. 2019), which is directionally consistent with our observations despite differences in exposure duration and developmental stage.

To further validate these transcriptional patterns, expression of selected heat‐responsive genes was examined by RT‐qPCR in the three strains. Consistent with the RNA‐seq results, *hsp‐16.2* was strongly induced following heat shock in the N2 Lab strain, whereas *hsf‐1* and *daf‐18* showed reduced expression and *hsp‐6* exhibited a decrease (Figure S2C). In contrast, all four genes showed upregulation in both the N2 Ancestral and Mexican strains, mirroring the strain‐specific expression patterns observed in the RNA‐seq data.

Finally, the N2 Lab–specific cluster of upregulated genes included *swan‐1* (involved in heat and osmotic stress responses; Ritterhoff et al. 2010), *eya‐1* (whose downregulation reduces heat and oxidative stress tolerance; Wang et al. 2014), the transcription factor *jmjc‐1* (a regulator of stress‐associated genes; Kirienko and Fay 2010), and *F08H9.3*, associated with desiccation resistance (Erkut et al. 2013). These findings point to a distinct heat‐stress‐associated expression pattern in the N2 Lab strain.

Overall, these results reveal conserved and strain‐specific transcriptional patterns under acute heat stress. The acute heat‐stress response was more similar between the Mexican and Ancestral strains than between either strain and N2 Lab, consistent with strain‐specific differences in transcriptional dynamics across genetic backgrounds (Jovic et al. 2017).

## 3 Discussion

Variation in environmental and laboratory history can influence how model organisms perform under different experimental conditions. Here, we compared life‐history traits, stress‐resistance phenotypes, and transcriptomic profiles among three *C. elegans* strains with distinct histories: a recently isolated Mexican sample, the N2 Ancestral lineage (a historically distinct lineage with substantially less propagation) (Sterken et al. 2015), and the contemporary N2 Lab strain. Our results show that these strains differ in lifespan, fertility, pathogen and oxidative stress resistance, thermotolerance, and gene‐expression patterns. The experimental conditions used in this study incorporate key ecologically relevant variables (including temperature, diet, and biotic and abiotic stressors) aiming to bridge laboratory observations to natural environments.

At the phenotypic level, the Mexican strain exhibited reduced lifespan and fertility under standard laboratory conditions but increased survival following *Pseudomonas aeruginosa* PA14 exposure, whereas the Ancestral strain showed higher oxidative stress resistance alongside reduced thermotolerance. These results indicate that strain differences are stressor‐specific rather than uniformly beneficial or detrimental. The reduced lifespan and fertility of the Mexican strain may reflect the fact that standard laboratory conditions are less favorable for a recently isolated strain. The lower fertility of the Mexican strain may also reflect multiple nonexclusive factors, including differences in sperm production, oocyte quality, embryonic viability, or responses to the monobacterial *E. coli* diet used in culture, as bacterial food sources can influence brood size and reproductive span (MacNeil et al. 2013; Stuhr and Curran 2020; Le et al. 2022). These differences may also reflect a trade‐off between traits that enhance survival outside the laboratory, such as stress tolerance or defensive responses, and traits favored under stable laboratory conditions, such as maximal reproductive output, though this interpretation remains speculative. These data should be interpreted as phenotypic differences under laboratory conditions, rather than direct evidence of reduced fitness in natural environments.

The Mexican strain carries the *npr‐1*215F allele and displayed the associated social feeding behavior, whereas the N2 Lab and Ancestral strains fed solitarily under our conditions. Variants at this locus have been associated with differences in susceptibility to bacterial pathogens, largely through behavioral effects on the time animals spend on the bacterial lawn (Reddy et al. 2009; Martin et al. 2017). We therefore do not attribute the increased PA14 survival of the Mexican strain to *npr‐1* genotype, and its basis remains to be determined.

The contrasting responses to paraquat and heat stress also point to stressor‐specific phenotypic variation. Because the N2 Lab and Ancestral strains derive from the same laboratory lineage and behaved similarly in our feeding assays, differences in paraquat sensitivity between them are unlikely to be explained by the loci underlying social feeding. Other factors, including unintended genetic drift accumulated during laboratory propagation or differences in baseline regulatory states, may contribute to the differences observed between strains under these stressors.

Despite the similarity of the transcriptional responses of the Mexican and Ancestral strains at 35°C, the Ancestral strain showed reduced thermotolerance, which became apparent only after 4 h of exposure (Figure 1G). Something to take into consideration is that transcriptional profiles were obtained after 2 h at 35°C, when the survival of both strains was still comparable, so the transcriptional differences underlying this divergence may not yet have been established at the time of sampling. Because the heat stress transcriptional response is dynamic (Jovic et al. 2017), profiling both strains at later exposure times would be needed to identify the genes involved. The reduced thermotolerance of the Ancestral strain may also reflect an antagonism between oxidative stress and heat stress responses rather than its geographic origin. Activation of the SKN‐1‐dependent oxidative stress response has been shown to reduce survival during heat stress, independently of HSF‐1 (Crombie et al. 2016), and the Ancestral strain, which was the most resistant to paraquat, also showed the highest baseline expression of SKN‐1 target genes such as *gst‐4* (Table S2). Although this correspondence is suggestive, it is based on transcript abundance rather than on direct measurement of pathway activity, and remains to be tested.

The transcriptomic analyses provided a complementary molecular layer to these phenotypic observations. Under standard conditions, the N2 Lab strain showed the most distinct global expression profile, whereas the Mexican and Ancestral strains were more similar to each other (a pattern also observed under acute heat stress). Additionally, standard laboratory conditions, including atmospheric oxygen levels and a monobacterial diet, may themselves represent an altered environment for non‐domesticated strains, and broad transcriptional differences may partly reflect responses to these conditions (MacNeil et al. 2013). These data should not be interpreted as evidence that laboratory propagation alone caused the observed differences; genetic variation, laboratory history, environmental history, and regulatory differences may all contribute, consistent with previous work showing that genetic background strongly influences gene‐expression profiles in *C. elegans* (Rockman et al. 2010; Snoek et al. 2017; Zhang et al. 2022; Bell et al. 2025).

Immune‐related genes were enriched among Mexican‐specific upregulated genes, consistent with the increased PA14 survival of this strain. For example, *lys‐7* has been implicated in defense against PA14, whereas reduced expression of *spl‐2* has been linked to decreased PA14 susceptibility (Evans et al. 2008; Fatin et al. 2017; Nasrallah et al. 2023). Pathogen resistance also varies according to both strain backgrounds and the specific pathogen tested (Schulenburg and Müller 2004; Balla et al. 2015; Lansdon et al. 2022). Oxidative stress‐related genes also differed among strains: the Mexican strain showed reduced expression of *ctl‐1*, whereas the Ancestral strain showed higher expression of *sod‐3*, *pah‐1*, and additional antioxidant‐related genes. However, because these RNA‐seq data were generated under control conditions, these patterns reflect baseline expression differences rather than direct stress‐induced responses, and integrating strain‐specific genomic information will be important to further resolve these contributions.

Among genes previously linked to lifespan regulation, none provides a straightforward explanation for the reduced lifespan of the Mexican strain. At 20°C, *daf‐2* and *skn‐1* were downregulated relative to N2 Lab to a similar extent in the Mexican and Ancestral strains (*daf‐2*, LFC = −3.04 and −2.65; *skn‐1*, LFC = −1.92 and −1.90; Table S2), and their upregulation was confined to heat stress, even though the two strains differ in lifespan in opposite directions. The two genes also predict opposite outcomes, since reduced insulin/IGF‐1 receptor (DAF‐2) signaling is associated with lifespan extension (Kenyon et al. 1993), whereas SKN‐1 promotes longevity (Tullet et al. 2017). *lipl‐4*, the only lifespan‐related gene upregulated exclusively in the Mexican strain, is a lysosomal lipase whose overexpression extends lifespan (Lapierre et al. 2011; Folick et al. 2015), yet the Mexican strain had the shortest median lifespan of the three strains (Figure 1B). Transcript levels of individual pathway components may therefore not be informative about pathway activity, as also suggested by the downregulation of *daf‐16*, *daf‐18*, and *skn‐1* in both the Mexican and Ancestral strains (LFC = −1.46 to −2.84; Table S2) alongside upregulation of downstream stress‐response targets such as *sod‐3* and *gst‐4* in the Ancestral strain; investigating whether these pathways contribute to the lifespan differences observed in this work would require direct experimental validation.

Cuticle‐ and chromatin‐related genes also emerged among strain‐specific expression differences. The shared upregulation of collagen genes in the Mexican and Ancestral strains is consistent with variation in collagen expression reported among *C. elegans* strains (Bell et al. 2025) and with evidence that cuticle‐related traits differ between backgrounds (Noble et al. 2020). The enrichment of chromatin‐related categories and differential expression of chromatin‐associated genes identify regulatory factors worth exploring in future work; the current observations should be considered descriptive rather than evidence for a direct epigenetic mechanism.

Following acute heat stress, all three strains activated a conserved core response, including canonical heat‐inducible genes such as *hsp‐16.2*, *hsp‐16.41*, and *hsp‐70* (Brunquell et al. 2016; Schreiner et al. 2019; Xu et al. 2023). Beyond this shared response, the Mexican and Ancestral strains shared a larger fraction of heat‐responsive DEGs with each other than with N2 Lab, and selected RT‐qPCR validations supported strain‐specific expression patterns for *hsf‐1*, *hsp‐6*, *hsp‐16.2*, and *daf‐18*, consistent with previous work showing that heat‐stress transcriptional dynamics and resilience vary across genetic backgrounds (Jovic et al. 2017, 2019). The broader set of heat‐responsive genes in the Ancestral and Mexican strains, including induction of *hsf‐1*, *hsp‐6*, and stress‐signaling components, is consistent with a more extensive transcriptional reorganization under acute stress in these strains, possibly reflecting a more inducible stress response compared to a more preadapted transcriptional state in the N2 Lab strain, though this interpretation remains to be tested.

Several aspects of this study warrant consideration when interpreting the results. The Mexican strain represents a single recently isolated sample, limiting generalization to broader *C. elegans* diversity. A more complete understanding of the observed differences will require integrating strain‐specific genomic variation with transcriptional data, and future epigenetic profiling (including chromatin accessibility and histone modification analyses) will be important to evaluate whether regulatory differences contribute to the transcriptional patterns described here. Yet, our findings contribute to a broader characterization of phenotypic and transcriptomic variation in *C. elegans* and highlight how strain history can be associated with measurable differences in experimental outcomes.

## 4 Materials and Methods

### 4.1 Nematode and Bacterial Strains

*Caenorhabditis elegans* laboratory strains used in this study were: wild type N2 (var. Bristol) and N2 Ancestral strain, originally frozen in 1969 and thawed in 1980, which, although cultivated in laboratory conditions, is estimated to be fewer than six generations from the original stock. Both strains were obtained in 2023 from the Caenorhabditis Genetics Center (CGC) and were thawed 2 weeks before each experiment. The VJV2 (Mexican) strain was isolated in Mexico City in June 2023 (this study). Strains were maintained on NGM plates, fed on *E. coli* OP50‐1 at 20°C (Stiernagle 2006) and synchronized by bleaching. All *C. elegans* strains were unfrozen from a new vial each month to prevent them from exceeding 10 generations from the original stock. The following bacterial strains were used: *Pseudomonas aeruginosa* strain PA14; and *E. coli* RNAi clones targeting *dpy‐11* and *pos‐1* from the Ahringer RNAi library (Kamath and Ahringer 2003). *C. elegans* protocols were approved by the IFC‐IACUC (protocol VVR191‐22).

### 4.2 Mexican *C. elegans* Isolation

Sampling was conducted in the gardens of Instituto de Fisiología Celular, UNAM, in México City (coordinates: 19.32840, −99.17846). Pieces of ripe pear were buried 1–2 cm deep in moist soil and collected after 1 week along with surrounding soil and debris. Wild isolates were processed as specified in Barrière and Félix (2014). Briefly, each sample was transferred to a 100 mm NGM plate seeded with *E. coli* lawn and sealed with parafilm. After 3–5 days, plates were screened for worm trails; adult worms were individually transferred to a 30 mm plate with food. After 3 days, the presence of double‐bulb pharynx, characteristic of *C. elegans* , was confirmed by microscopy. For individuals showing this feature, species identity was confirmed by PCR amplicon of the ITS2 gene (Internally Transcribed Spacer between 5.8S and 28S rDNA genes) using RHAB1350F (5′‐TACAATGGAAGGCAGCAGGC) and RHAB1868R (5′‐CCTCTGACTTTCGTTCTTGATTAA) primers that in *C. elegans* gives a 557 bp amplicon. Samples yielding this product were subjected to a second PCR with primers 5.8S‐1 (5′‐CTGCGTTACTTACCACGAATTGCARAC) and KK‐28S‐22 (5′‐CACTTTCAAGCAACCCGAC), which results in a 777 bp amplicon specific for *C. elegans* . The amplicon was sequenced by Sanger and analyzed by NCBI/BLAST tool.

### 4.3 Lifespan Assay

Assays were conducted using synchronized populations of L4‐stage worms. For each strain, approximately 180 worms were distributed in three 30 mm NGM plates and monitored daily for survival until all individuals had died. During the first 5–7 days, worms were transferred to fresh plates daily. After this period, worms were transferred every other day to ensure food supply. Worms that exhibited bagging or crawled off the plate were censored from the analysis. Worms were considered dead if they failed to respond to gentle prodding with a platinum wire. Each lifespan assay was repeated in three independent biological replicates.

### 4.4 *Pseudomonas aeruginosa* PA14 Survival Assay

A fast‐killing *Pseudomonas aeruginosa* PA14 assay was performed as described by Liu et al. (2024), with minor modifications. Briefly, PA14 cultures were initiated from fresh LB plates (less than 1 month old). Prior to the assay, bacteria were grown overnight in LB broth at 37°C for no longer than 16 h. 150 μL of the overnight culture were spread evenly onto 33 mm NGM plates, ensuring complete surface coverage. Plates were allowed to dry and incubated upside down at 37°C for 15 h followed by 6 h incubation at 26°C. Approximately 50 synchronized L4‐stage worms were transferred to each plate (3 plates per strain) and maintained at 26°C. Worm mortality was assessed at intervals of 8–15 h over ~50 h. The assay was repeated in two independent biological replicates.

### 4.5 Fertility Assays

Synchronized L4‐stage worms were individually placed on 33 mm NGM plates seeded with *E. coli* . Worms were transferred to fresh plates every 24 h for 6 days. Hatched embryos were counted 48 h after removal of parental hermaphrodite. This procedure was repeated until the progeny of ≥ 18 parental worms were analyzed. Total fertility was calculated by summing the number of hatched embryos (L1 larvae) produced daily by each worm.

### 4.6 35°C Survival Assay

One‐day adult worms (approximately 25 per plate) were placed on 33 mm NGM plates seeded with *E. coli* . The experiment was conducted in triplicate, with three plates per strain per hour. Plates were incubated in a water bath at 35°C; three plates per strain were removed each hour from 4 to 9 h. After removal, plates were transferred to 20°C for recovery. Survival was assessed 12 h after the final time point by scoring live and dead worms. Each survival assay was repeated in three independent biological replicates.

### 4.7 Oxidative Stress Resistance Assay

Synchronized L4‐stage worms were exposed to oxidative stress using paraquat. Approximately 30 individuals per strain were placed into each well of a 96‐well plate containing 300 μL of M9 buffer supplemented with *E. coli* and 250 mM of paraquat (along with 0 mM PQ controls). Each strain was tested in duplicate. Worm mortality was recorded every 30 min over the course of the experiment. Each survival assay was repeated in two independent biological replicates.

### 4.8 Chemotaxis

Synchronization was performed as described by Remy and Hobert (2005): day 1 adults were allowed to lay approximately 300 eggs in 60 mm NGM plates with food. Adults were discarded by gently washing the plates with M9; remaining eggs were incubated at 20°C until they reached the young adult stage. Chemotaxis assays were conducted as in Bargmann et al. (1993): approximately 200 worms were placed in the center of a 100 mm plate containing the test odorants at one end of the plate and the vehicle, as a control, at the other. NaN_3_ was placed on both ends to paralyze the animals. The odorant concentrations and vehicles used were the following: benzaldehyde 0.33% diluted in H20; butanone 0.01% diluted in 100% ethanol; isoamyl alcohol 1% diluted in 100% ethanol; and octanol 0.1% diluted in 100% ethanol. Chemotaxis index was calculated with the following formula: $\mathrm{CI}=\left(N\;\text{odorant}-N\;\text{control}\right)/N\;\text{total}.$

### 4.9 RNA Interference

RNAi experiments targeting *pos‐1* and *dpy‐11* genes were conducted using *E. coli* HT115 strain engineered to express double‐stranded RNA (Kamath and Ahringer 2003). Bacteria were cultured overnight at 37°C in LB broth containing ampicillin (100 mg/mL) and tetracycline (12.5 mg/mL). NGM plates with ampicillin (10 μg/mL) were overlaid with IPTG at indicated concentration to induce dsRNA expression. Plates were left overnight at room temperature protected from light. For *pos‐1* RNAi, one L4‐larva was placed on each RNAi plate and progeny were monitored daily for 6 days. To assess gene silencing, hatched and unhatched progeny were counted. For *dpy‐11*, one gravid worm was placed per plate for ~4 h to lay eggs and synchronize the population. Scoring was counted 72 h later to assess dumpy phenotype. All RNAi experiments were performed at 20°C.

### 4.10 Reverse Transcription and Quantitative PCR

cDNA was synthesized from total RNA using the SuperScript III reverse transcription kit (Invitrogen) with oligo(dT) primers, following the manufacturer's instructions. Quantitative PCR was performed with the KAPA SYBR FAST qPCR Master Mix (2×) in a final reaction volume of 10 μL, using cDNA diluted 1:10, on a Bio‐Rad CFX384 real‐time PCR system. Expression of each gene was normalized to act‐1 using the 2^‐ΔCt^ method. Primer sequences are listed in Table S3.

### 4.11 RNA‐Seq Library Preparation

Total RNA was isolated from synchronized populations harvested at day 1 of adulthood (72 h post‐hatching). For each strain and temperature condition, independent biological replicates were generated, each consisting of an independently grown population of synchronized worms. Following quality control, sample sizes were as follows: at 20°C, N2 Lab (*n* = 2), N2 Ancestral (*n* = 2), and Mexican strain VJV2 (*n* = 3); at 35°C, N2 Lab (*n* = 3), N2 Ancestral (*n* = 2), and VJV2 (*n* = 3). To control for potential differences in developmental rate among strains, developmental timing was assessed by monitoring vulval morphology during the L4 stage, and all strains reached the characteristic L4 vulval morphology at the same chronological time under our experimental conditions. Worms were collected with M9 buffer and washed twice to remove bacteria. Three hundred microliters of TRIzol were added to each sample, followed by eight cycles of liquid nitrogen freezing and thawing at 37°C to ensure lysis. This was followed by four cycles of vortexing (20 s at maximum speed, then 20 s on ice). Samples were centrifuged at 10,000 × g for 1 min, and RNA was purified using the Direct‐zol RNA MicroPrep kit (Zymo, R2062) following the manufacturer's instructions. RNA integrity and concentration were evaluated using a 2100 Bioanalyzer (Agilent), and only samples with RIN > 8 were selected for library preparation. All samples were processed in parallel: RNA extractions, library preparations and sequencing were performed concurrently for all strains and temperature conditions, with no differences in extraction, processing or sequencing dates between N2 and the other strains. All libraries were sequenced together in a single run on a NextSeq 500 system (Illumina) using paired‐end 75‐bp reads, minimizing potential batch effects.

### 4.12 RNA‐Seq Data Analysis

Data quality was assessed using FastQC. Alignments of paired‐end samples were generated using STAR (version 2.7.11b) with default parameters, except for ‐‐sjdbOverhang 149 and ‐‐genomeSAindexNbases 12, using the *Caenorhabditis elegans* reference genome WBcel235 obtained from the Ensembl portal. Alignment quality was evaluated using STAR Log.final.out files, including the percentage of uniquely mapped reads, the percentage of reads mapped to multiple loci, and the percentage of reads unmapped due to excessive mismatches. Raw gene‐level count matrices were generated using FeatureCounts with flags ‐p and ‐a. Differential gene expression analysis was performed using DESeq2 (v1.42.1). For strain‐level comparisons, counts were modeled using the design formula ~ strain, and differential expression was assessed using Wald tests with explicit contrasts between strains. To evaluate heat‐dependent transcriptional responses, differential expression analyses were performed separately within each strain using the design formula ~ condition, with contrasts defined between temperature conditions. Genes with an adjusted *p* value < 0.05 and an absolute log2 fold change > 2 were considered differentially expressed, unless stated otherwise. Differential expression results for all comparisons have been deposited in GEO (GSE301219) as Supporting Information files. Because RNA‐seq reads from all strains were aligned to the Bristol N2 reference genome, expression estimates for genetically divergent strains may be conservatively biased for highly polymorphic genes. Alignment metrics indicated low mismatch‐related unmapped read rates and comparable mapping performance across strains under control conditions, suggesting no major impairment of read alignment. However, such global metrics do not exclude the possibility of more subtle biases in highly divergent regions.

Pathway enrichment analyses were performed using WormCat (Holdorf et al. 2020) with a *p*‐value cutoff of 0.05 to define enriched categories. We note that WormCat does not currently support user‐defined background gene sets; therefore, enrichment statistics are computed relative to the full set of annotated *C. elegans* genes rather than the subset of genes tested by DESeq2. As a result, enrichment results should be interpreted qualitatively, as descriptive summaries of functional themes among DEGs, rather than as precise estimates of pathway overrepresentation. Heatmaps were generated using DESeq2 variance‐stabilizing transformation normalized counts and the R package Pheatmap. Volcano plots and transcript expression patterns were generated using RStudio (v4.3.3) using custom code available at [https://github.com/jaq77/Mexa‐C.‐elegans.git]. Genes lists for heatmaps were obtained from amigo.geneontology.org/amigo (Ashburner et al. 2000; Gene Ontology Consortium et al. 2023), using the Advanced annotation search, with the following fillers: Organism: *Caenorhabditis elegans* ; Type: gene; GO class (including “regulates”). GO class for: (1) Lifespan = Insulin, Energy metabolism, Determination of adult lifespan; (2) Reproduction = Spermatogenesis, Spermatid development, Spermidine biosynthetic process, Amoeboid sperm motility, Flagellated sperm motility, Sperm flagellum, Positive regulation of amoeboid sperm motility, Sperm ejaculation, Sperm entry, Spermathecum morphogenesis, Spermidine metabolic process, Gonad development, Gonad morphogenesis, Positive regulation of gonad development, Gonadal mesoderm development, Male gonad development, Female gonad development, Positive regulation of female gonad development, Oogenesis, Positive regulation of oogenesis, Oocyte maturation, Oocyte development, Positive regulation of oocyte development, Oocyte growth, Regulation of meiotic cell cycle process involved in oocyte maturation, Oocyte morphogenesis, Positive regulation of oocyte maturation, Negative regulation of oocyte maturation, Regulation of oocyte development; (3) ROS = Response to oxidative stress, Positive regulation of response to oxidative stress, Cellular response to oxidative stress, Negative regulation of response to oxidative stress, Regulation of response to oxidative stress, Cell redox homeostasis, some genes from Rafikova et al. (2020); (4) Heat stress = Response to heat, Heat acclimation, Heat shock protein binding, Cellular response to heat, Negative regulation of cellular response to heat, Positive regulation of cellular response to heat, Regulation of cellular response to heat, Cellular heat acclimation; (5) Immune system = Immune response, Antibacterial innate immune response, Defense response to gram positive bacterium, Defense response to gram negative bacterium; (6) Epigenetic = Epigenetic regulation, Chromatin remodeling, Chromosome organization, Histone binding, Histone acetyltransferase activity, Histone deacetylase activity, Methylated histone binding.

## Author Contributions

**Jaqueline Hersch‐González:** conceptualization (equal), data curation (lead), formal analysis (lead), investigation (equal), methodology (lead), writing – original draft (lead), writing – review and editing (equal). **Nallely Cano‐Domínguez:** methodology (supporting), project administration (equal). **Mariana Zurita‐León:** methodology (supporting), writing – review and editing (supporting). **Francisco Pinta‐Castro:** methodology (supporting), writing – review and editing (supporting). **Maribel Soto‐Nava:** methodology (supporting). **Santiago Avila‐Rios:** resources (equal), supervision (supporting), writing – review and editing (supporting). **Victor Julian Valdes:** conceptualization (equal), data curation (equal), funding acquisition (lead), resources (equal), supervision (equal), writing – original draft (equal), writing – review and editing (equal).

## Funding

This work was supported by Dirección General de Asuntos del Personal Académico, Universidad Nacional Autónoma de México, PAPIIT‐UNAM IN217824. Secretaría de Ciencia, Humanidades, Tecnología e Innovación (SECIHTI), Mexico, CBF‐2025‐I‐2275.

## Conflicts of Interest

The authors declare no conflicts of interest.

## Supporting information


**Figure S1:** Species confirmation, RNA interference response and chemotaxis assays in the VJV2 (Mex) strain. (A) Map of the ITS2 gene (Internally Transcribed Spacer between 5.8S and 28S rDNA genes) of *C. elegans* and PCR amplification of ITS2 fragments: 557 bp using RHAB1350F and RHAB1868R primers; 777 bp with 5.8S‐1 and KK‐28S‐22 primers. (B) Alignment of the Sanger‐sequenced 777 bp amplicon using the NCBI/BLAST, showing 100% identity. (C) Feeding behavior: Left, the N2 Lab strain shows the typical *npr‐1*215V “solo” feeding phenotype; Right, the VJV2 (Mex) strain shows the *npr‐1*215F “social” feeding phenotype. (D) RNA interference (*RNAi*) assay. Embryonic lethality was assessed by silencing the *pos‐1* gene with 0.01 mM IPTG. The *Dumpy* phenotype was induced by silencing the *dpy‐11* gene using 7 mM and 14 mM IPTG. No differences were observed between the three strains in either assay. Statistically significance was assessed by two‐way ANOVA with multiple comparisons. Graphs show representative experiments with consistent trends across 3–4 independent replicates. (E) Chemotaxis assays toward benzaldehyde (0.33%), butanone (0.01%), isoamyl alcohol (1%) and octanol (0.1%) showed no significant differences among the three strains. Statistical significance was assessed by one‐way ANOVA with multiple comparisons.

**Figure S2:** Principal component analysis of RNA‐seq at 20°C and 35°C and RT‐qPCR validation of selected genes under heat stress. (A) Principal component analysis (PCA) of normalized RNA‐seq expression values shows clustering of samples by strain at 20°C. Each point represents an independent biological replicate (N2 Lab, *n* = 2; Ancestral, *n* = 2; VJV2 (Mex), *n* = 3). Percent variance explained by each principal component is indicated on the axes. (B) PCA of normalized RNA‐seq expression values at 35°C. Each point represents an independent biological replicate (N2 Lab, *n* = 3; Ancestral, *n* = 2; VJV2 (Mex), *n* = 3). Percent variance explained by each principal component is indicated on the axes. (C) Relative expression by RT‐qPCR of *hsf‐1*, *hsp‐6*, *hsp‐16.2*, and *daf‐18* genes normalized to *act‐1*. Each point represents an independent biological replicate (N2 Lab, *n* = 2; Ancestral, *n* = 2; VJV2, *n* = 3).

**Table S1:** STAR mapping summary.

**Table S2:** Differential gene expression log_2_ fold change (LFC) values (all with a *p*‐adjusted value ≤ 0.05).

**Table S3:** Primers used for qPCR analysis.

## Data Availability

The data underlying this article is available in the Gene Expression Omnibus repository under the accession number GSE301219. All the required data are uploaded as Supporting Information.
