# Supplementary material for: Life‐History, Stress‐Resistance, and Transcriptomic Variation Among a Mexican Caenorhabditis elegans Isolate and Contemporary and Ancestral N2 Lineages
Source: Ecol Evol. 2026 Sep 30;16(10):e74378. doi: 10.1002/ece3.74378 (PMC13627867; doi:10.1002/ece3.74378)
Supplement: Supplementary file 1 [file ECE3-16-e74378-s001.docx]

**Table S2** Differential gene expression log_2_ fold change (LFC) values (all with a *P*-adjusted value ≤ 0.05)

|  |  | **20 ºC** |  |  | **35 ºC** |  |
| --- | --- | --- | --- | --- | --- | --- |
| **Gene name** | **N2 Ancestral vs. N2 Lab** | **VJV2 (Mex) vs. N2 Lab** | **VJV2 (Mex) vs. N2 Ancestral** | **N2 Lab** | **N2 Ancestral** | **VJV2 (Mex)** |
| *C04G2.10* | - | -1.78 | -2.42 | - | - | - |
| *C25F9.4* | -2.10 | -8.52 | -6.42 | -4.97 | 1.87 | - |
| *C25F9.5* | -2.82 | -8.67 | -5.84 | -6.42 | 2.40 | - |
| *cbp-2* | - | -9.91 | -8.85 | -3.93 | - | - |
| *cct-2* | -1.66 | - | - | - | - | - |
| *cct-4* | -1.56 | - | - | - | - | - |
| *cct-8* | -1.88 | - | - | - | 1.73 | - |
| *ctl-1* | - | -8.36 | -7.99 | -1.62 | - | - |
| *daf-16* | -1.65 | -1.46 | - | - | 1.92 | 1.70 |
| *daf-18* | -2.49 | -2.84 | - | - | 2.49 | 3.27 |
| *daf-2* | -2.65 | -3.04 | - | - | 3.38 | 3.90 |
| *dnj-25* | -1.59 | - | - | - | 2.00 | 1.86 |
| *dnj-27* | 1.51 | - | - | 2.95 | -1.63 | - |
| *dnj-5* | -1.94 | -2.40 | - | - | 2.01 | 2.69 |
| *elt-3* | 1.66 | 1.14 | - | - | - | - |
| *endu-2* | -2.02 | - | 1.60 | -3.41 | 2.19 | - |
| *F08H9.3* | - | - | - | 8.45 | - | - |
| *F44E5.5* | - | - | - | 5.63 | 5.84 | - |
| *gpx-5* | 1.94 | - | - | - | -1.95 | -1.60 |
| *gst-10* | 1.72 | - | - | -1.80 | -3.46 | -1.94 |
| *gst-4* | 1.75 | - | - | - | -2.64 | -2.15 |
| *hil-2* | 2.28 | 2.01 | - | 2.23 | -2.53 | -1.80 |
| *hsf-1* | -1.83 | -2.01 | - | - | 1.97 | 2.32 |
| *hsp-1* | -2.11 | - | - | -2.32 | 1.97 | - |
| *hsp-110* | -2.56 | -1.53 | - | - | 3.63 | 2.31 |
| *hsp-12.2* | 3.08 | 3.26 | - | 2.25 | -2.20 | -3.47 |
| *hsp-12.3* | - | 3.47 | 3.03 | - | - | -1.71 |
| *hsp-12.6* | - | - | - | 2.38 | 3.61 | - |
| *hsp-16.2* | - | - | - | 14.21 | 9.75 | 9.27 |
| *hsp-16.41* | - | - | - | 17.56 | 8.79 | 8.01 |
| *hsp-6* | -2.51 | - | - | -2.00 | 2.72 | 1.50 |
| *hsp-60* | -2.10 | - | 1.70 | -2.74 | 2.20 | - |
| *hsp-70* | - | - | - | 12.19 | 10.88 | 11.05 |
| *hsp-90* | -1.62 | - | - | - | 1.58 | - |
| *jmjc-1* | - | - | - | 3.06 | -2.64 | -1.62 |
| *jnk-1* | - | - | - | - | - | -2.10 |
| *K07F5.16* | - | - | - | 2.78 | - | - |
| *lipl-4* | - | 1.86 | 2.63 | - | - | - |
| *lys-7* | - | 3.09 | 2.24 | - | - | -2.06 |
| *M04C3.1* | -2.60 | -10.54 | -7.94 | -3.50 | 2.95 | - |
| *M04C3.2* | -2.62 | -7.49 | -4.87 | -7.11 | 2.76 | - |
| *mtl-2* | - | - | - | -3.91 | -3.40 | -2.61 |
| *pah-1* | 2.14 | - | - | - | -2.12 | -1.58 |
| *rme-8* | -2.65 | -2.18 | - | -1.90 | 2.93 | 2.47 |
| *rpn-2* | -1.84 | - | - | - | 1.78 | - |
| *skn-1* | -1.90 | -1.92 | - | - | 1.90 | 2.02 |
| *sod-3* | 2.68 | - | -2.26 | - | -2.09 | - |
| *spl-2* | - | -2.58 | -2.67 | -4.53 | -2.86 | - |
| *swan-1* | - | - | - | 2.82 | - | - |
| *ttx-1* | 2.07 | 2.48 | - | - | -2.41 | -2.89 |
| *uba-1* | -2.48 | -1.74 | - | - | 2.44 | 1.97 |
| *Y17G9B.8* | -2.33 | - | 3.32 | - | 2.28 | - |

***Complete DESeq2 information can be found in supplemental material.**
