# Supplementary material for: Life‐History, Stress‐Resistance, and Transcriptomic Variation Among a Mexican Caenorhabditis elegans Isolate and Contemporary and Ancestral N2 Lineages
Source: Ecol Evol. 2026 Sep 30;16(10):e74378. doi: 10.1002/ece3.74378 (PMC13627867; doi:10.1002/ece3.74378)
Supplement: Supplementary file 2 [file ECE3-16-e74378-s003.docx]

**Table S1** STAR mapping summary.

| sample | input_reads | uniq_reads_n | uniq_reads_pct | multi_reads_pct |
| --- | --- | --- | --- | --- |
| Anc_c1Log.final.out | **24133289** | **21151111** | **87.64%** | **1.86** |
| Anc_c2Log.final.out | **41134363** | **35165333** | **85.49%** | **3.08** |
| Anc_t2Log.final.out | **29618213** | **24901603** | **84.08%** | **3.75** |
| Anc_t3Log.final.out | **34991869** | **30541090** | **87.28%** | **3.69** |
| Mex_c1Log.final.out | **25665645** | **22173970** | **86.40%** | **2.70** |
| Mex_c2Log.final.out | **28610685** | **22173469** | **77.50%** | **4.92** |
| Mex_c3Log.final.out | **21664149** | **18636291** | **86.02%** | **2.11** |
| Mex_t1Log.final.out | **24319123** | **20863246** | **85.79%** | **4.42** |
| Mex_t2Log.final.out | **26440349** | **22891328** | **86.58%** | **3.36** |
| Mex_t3Log.final.out | **26168642** | **22559196** | **86.21%** | **2.88** |
| N2_c2Log.final.out | **30486375** | **26880310** | **88.17%** | **3.07** |
| N2_c3Log.final.out | **32156301** | **28199892** | **87.70%** | **2.67** |
| N2_t1Log.final.out | **27316780** | **19791982** | **72.45%** | **21.16** |
| N2_t2Log.final.out | **26922854** | **20883216** | **77.57%** | **14.56** |
| N2_t3Log.final.out | **26735880** | **19767833** | **73.94%** | **15.96** |
