# Supplementary material for: Life‐History, Stress‐Resistance, and Transcriptomic Variation Among a Mexican Caenorhabditis elegans Isolate and Contemporary and Ancestral N2 Lineages
Source: Ecol Evol. 2026 Sep 30;16(10):e74378. doi: 10.1002/ece3.74378 (PMC13627867; doi:10.1002/ece3.74378)
Supplement: Supplementary file 3 [file ECE3-16-e74378-s005.docx]

**Table S3** Primers used for qPCR analysis.

| Name | Sequence | Scale | Purification |
| --- | --- | --- | --- |
| act-1_FW_RT | CTCTTGCCCCATCAACCATGA | 25nm | STD |
| act-1_RV_RT | TTTGTGCAAGTTGACGAAGTTGT | 25nm | STD |
| hsf-1_FW_RT | ATGCAGCCAGGATTGTCGAA | 25nm | STD |
| hsf-1_RV_RT | GCACGTTTTGAGTTGGGTCC | 25nm | STD |
| hsp-6_FW_RT | AGCTCACCAGAGCCAAGTTC | 25nm | STD |
| hsp-6_RV_RT | TTACGGCATGGCTCAATGGT | 25nm | STD |
| hsp-16.2_FW_RT | TCAACGTTCCGTTTTTGGTG | 25nm | STD |
| hsp-16.2_RV_RT | TCAGAAGACTCAGATGGAGAGAT | 25nm | STD |
| daf-18_FW_RT | CTCAGCCGTACATCGGAGAC | 25nm | STD |
| daf-18_RV_RT | TCGCATCGGCGTTTCATTTC | 25nm | STD |
